# Comprehensive Review of Hair Dyes: Physicochemical Aspects, Classification, Toxicity, Detection, and Treatment Methods

**DOI:** 10.1021/acsomega.5c01576

**Published:** 2025-06-27

**Authors:** João Carlos de Souza, Elisa Raquel Anastácio Ferraz Avelino, Guilherme Garcia Bessegato, Rogério do Carmo Gonçalves da Costa, Patricia Alves Carneiro, Danielle Palma de Oliveira, Maísa Azevedo Beluomini, Juliana Ferreira Brito, Maria Valnice Boldrin Zanoni

**Affiliations:** a Faculty of Philosophy, Sciences, and Letters at Ribeirão Preto (FFCLRP), Department of Chemistry, University of São Paulo (USP),Avenida Bandeirantes, 3900, Ribeirão Preto, São Paulo State 14040-901, Brazil; b Institute of Chemistry, Department of Analytical, Physical-Chemical and Inorganic Chemistry, São Paulo State University (UNESP), Rua Professor Francisco Degni, 55, Araraquara, São Paulo State 14800-060, Brazil; c Faculty of Pharmaceutical Sciences, University of São Paulo (USP), Ribeirão Preto Avenida Bandeirantes, 3900, Ribeirão Preto, São Paulo State 14040-901, Brazil; d Universidade Tecnológica Federal do Paraná (UTFPR), Dois Vizinhos Campus Estrada para Boa Esperança km 04, Dois Vizinhos, Paraná State 85660-000, Brazil; e Department of Chemistry, Institute of Exact Sciences (ICEx), Federal Fluminense University (UFF), Rua Desembargador Ellis Hermydio Figueira, s/n, Volta Redonda, Rio de Janeiro State 27213-145, Brazil; f School of Agricultural and Veterinary Sciences, São Paulo State University (UNESP), Via de Acesso Prof. Paulo Donato Castellane s/n, Jaboticabal, São Paulo State 14884-900, Brazil

## Abstract

Hair dyes have been a significant facet of human culture and identity, evolving from ancient natural pigments to complex synthetic formulations. This comprehensive review delves into the multifaceted dimensions of hair dyes, examining their physicochemical properties, classification, toxicity, detection, and treatment methods. The review explores the historical progression and contemporary advancements in hair dye technologies, highlighting the persistent challenges posed by their environmental and health impacts. It categorizes hair dyes into vegetable, mineral, and synthetic types, further subdividing synthetic dyes into temporary, semipermanent, and permanent categories based on their durability and application methods. This review also discusses the toxicological concerns associated with hair dyes, emphasizing the acute and chronic health risks posed by ingredients such as *p*-phenylenediamine (PPD) and its derivatives. Analytical methods for detecting and quantifying hair dyes in various matrices are evaluated, showcasing techniques such as chromatography and spectrophotometry. Furthermore, the review addresses the environmental implications of hair dye disposal, focusing on innovative treatment approaches, including advanced oxidation processes and bioremediation strategies. By synthesizing the last 20 years of the literature, this review provides a balanced perspective on the benefits and risks associated with hair dyes, offering insights into future research directions and sustainable practices to mitigate their adverse effects on human health and the environment.

## Graphical abstract

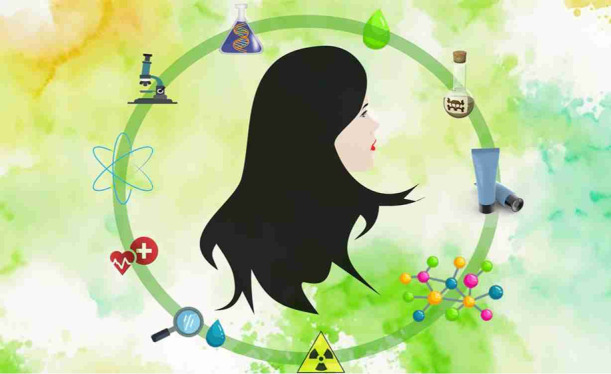

## 1 Introduction

Since the dawn of civilization, humans have dedicated part of their care to their health and appearance. Part of this care has been focused on hair because, in addition to reflecting a person’s identity and personality, it has an important psychological and cultural meaning that is fundamental to the construction of the image and conceptual identification of men and women in the society in which they live.^1–6^ The length, color, texture, and shape of hair are essential to build an individual’s physical appearance and self-perception and can be changed as desired to reflect how they want to be seen by others.^5^ Since ancient times, countless products and techniques have been developed and continue to be developed to achieve the most diverse changes and alterations in the physical characteristics of hair.^4^

Changing hair color is one of the oldest adornments in human activity, dating back thousands of years.^7^ In Egypt, paleontologists have found mummies about 4000 years old with hair dyed with henna.^7–9^ During the Roman Empire, men used combs soaked in a solution of lead sulfide and vinegar or exposed their hair to sulfurous vapors to darken or restore the natural color of gray hair gradually.^7–9^ On the other hand, women from the same civilization used to apply lye (caustic soda) and expose their hair to the sun to lighten it, imitating the blonde hair color of northern enslaved Europeans.^7–9^

From the 19th century to the present day, this approach to hair darkening has been carried out and marketed through the sale of various kits and products, including an aqueous solution of lead acetate containing a small amount of sulfur in suspension for daily application.^7–10^ However, various plant extracts are also still used to dye hair, such as walnut (*Juglans regia*), used to dye hair brown; chamomile (*Matricaria recutita*), used to dye hair yellow; and henna combined with indigo (*Indigofera suffruticosa*) to dye hair dark black.^7–10^

However, new hair dyeing products and processes have been developed with technological advances and the discovery of new compounds. In this sense, the oxidative hair dyeing process stands out, which has been practiced for over 150 years and evolved from the observation of the properties of *p*-phenylenediamine (PPD) by the chemist Dr. August Wilhelm Von Hofmann in 1863.^7,9–12^ PPD is a colorless compound that produces brown coloration when exposed to an oxidizing medium (atmospheric oxygen or through an oxidizing agent).^7–10^

The discovery of the properties of PPD allowed Monnet to obtain the first patent related to hair dyeing by oxidative processes in 1883 through the use of PPD and *p*-toluenediamine (PTD) as precursors for the formulation of hair dyes.^7,9,11,12^ Thus, with increasing knowledge of its potential, PPD began to be widely used and is part of the composition of practically all current hair coloring products.^7–12^ Between 1888 and 1897, the brothers and chemists Hugo and Ernst Erdmann further expanded the range of compounds that could be used in hair coloring; among them, it is possible to highlight *p*-aminophenol (PAP) and its derivatives, *N*-alkyl and *N*-phenyl-*p*-phenylenediamines, 4,4′-diaminodiphenylamine, and 1,5-diamino and 1,5-dihydroxynaphthalene.^11,13^ Their patents also stand out for the advantages of using hydrogen peroxide as an oxidizing agent in the dyeing process, which is an important chemical component that helps open the hair cuticle during coloring procedures and which aims to depigment the current hair color so that another color can be obtained.^11,13^ However, synthetic hair dyes only gained acceptance after the invention of the first commercial brand of synthetic hair dye, called Aureole, in 1909 by Eugène Schueller, a chemist and founder of the L’Oreal company.^7,14,15^

The hair care segment is booming and represents a large share of the global cosmetics market, around 22%, second only to the skin care category.^16^ On the other hand, during the COVID-19 pandemic, the cosmetics segment, along with other industrial sectors, was severely affected, with a drastic drop in consumption and consequently a weak commercial performance due to the lockdown restrictions that changed the habits of thousands of consumers worldwide.^17^ In 2022, the cosmetics industry recovered partially, with revenues of approximately 86 billion dollars worldwide and prospects of increasing to over 104 billion dollars by 2028.^16^

Even though there is an economic appeal from the hair dye industry, synthetic hair dyes typically exhibit moderate acute toxicity properties, with most of their main ingredients associated with allergies and dermatitis risks,^7,18,19^ depending on the ingredients that make up their formulations, such as precursors, couplings, and additives.^7^ The toxicological potential of hair dyes is related not only to the initial compounds used in the composition of the formulations of these products but also to secondary products and byproducts generated by oxidation, polymerization, and coupling reactions during hair dyeing. The class of aryldiamines used in hair dyes, including PPD, PTD, and their derivatives, has become a growing concern for health and environmental agencies worldwide.^7,20^ Some aromatic amines used in synthetic dye formulations or formed through biodegradation and partial degradation of these dyes are biologically active substances that can be absorbed percutaneously,^21,22^ potentially producing mutagenic or carcinogenic effects.^21–24^ With the rising global usage of hair dyes and expanding market influence, these products have emerged as a pressing public health concern, demanding an urgent assessment of their toxicity potential and environmental impact. Despite these concerns, the toxicity of hair dyes and their ingredients remains insufficiently investigated, with conflicting data regarding the actual risks to consumers.^9,25^

Despite the well-documented socioeconomic benefits and implications of this global trend, significant knowledge gaps persist regarding the fundamental mechanisms of the dyeing process and compound fixation in hair fibers and the actual environmental impact and health consequences for consumers.^26^ Moreover, the available data on these aspects often present discrepancies and conflicting information, primarily due to limitations in accurately reproducing the hair dyeing process and associated mechanisms, including oxidation, polymerization, transition, and metabolization of these compounds. Consequently, hair dyes are classified among Personal Care Products (PCPs), which have emerged as a critical group of contaminants of emerging concern.^27^

This comprehensive review aims to enhance the understanding of the hair coloring process by synthesizing current literature on multiple aspects of hair dyes. The review encompasses analytical methods for dye characterization, formulation compounds, and their reaction products while examining toxicological studies, degradation pathways, treatment approaches, and environmental and human health implications.

## 2 General Physical–Chemical Characteristics of Hair Dyes

Hair dyes can be classified according to their origin into three main categories: (1) vegetable dyes, derived from plants or plant parts (e.g., henna, chamomile, and cinchona); (2) mineral or metallic dyes, utilizing specific minerals or metallic salts for gradual hair lightening or darkening (e.g., silver nitrate, lye, and lead salts); and (3) synthetic dyes, produced artificially through the combination of one or more synthetic compounds. Synthetic dyes are further subdivided into temporary, semipermanent, and permanent categories based on their wash resistance and durability.^2,7,9,10,28–30^

### 2.1 Temporary Hair Dyes

The temporary dyes present low toxicity to humans, culminating in wide use by the industry for dyeing wool, silk, cotton, paper, food, and hair dyes, mainly to hide gray hair or to obtain more vibrant hair colors.^10,31^ They are generally marketed in the form of shampoos, conditioners, lotions, and gels with different color shades since those dyes do not require opening the cuticle to penetrate or diffuse into the structure of the hair fiber (cortex) and are only deposited on the external part of the hair shaft, therefore eliminating the need of ammonia or oxidizing agent use, unlike semipermanent and permanent dyes.^10,31^ For this reason, its color remains in the hair for only a few days and is easily removed by simply washing the hair.^32–34^

Temporary dyes are composed of basic or acidic dyes with high molecular weight and high solubility in water.^32–34^ Figure 1A shows the chemical structures of some compounds considered temporary acidic or basic dyes used in hair coloring. Acid dyes are anionic dyes fixed to the hair fiber by ionic bonds between the anionic groups of the pigment and the cationic groups of the amino acid residues present on the hair strand cuticle.^7,25^ Figure 1B illustrates the fixation of the temporary dye Acid Red 17 on a hair strand. On the other hand, basic dyes are cationic dyes that bind to the hair fibers through electrostatic interactions of the cationic groups in their structure and the anionic groups of the amino acid residues in the hair fiber cuticle.^7,25^ Figure 1C illustrates the temporary Basic Red 26 fixation on the hair fiber.

**Figure 1:**
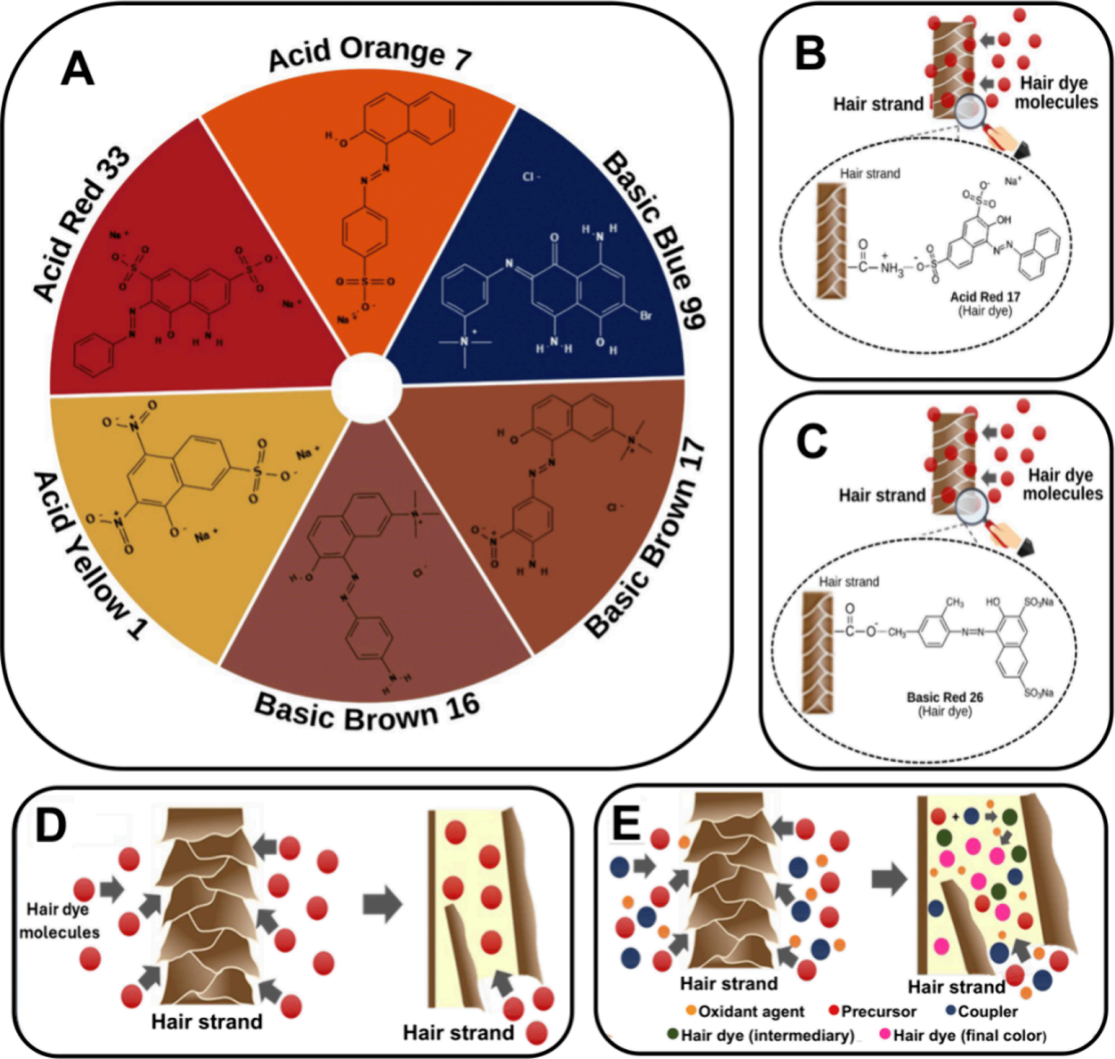
Acidic and basic temporary dyes commonly used in hair coloring (A), fixation mechanism of the temporary dyes Acid Red 17 (B) and Basic Red 26 (C), fixation mechanism of semipermanent hair dyes (D), and dyeing process of semipermanent hair dyes inside the hair fiber (E).

### 2.2 Semipermanent Hair Dyes

The other type of synthetic dye, semipermanent hair dye, also known as hair toner, is responsible for a 10% share of the dye market worldwide, found in shampoos, lotions, and sprays with different colors and shades.^10,25,31^ They provide a quick change in hair tone without drastically interfering with the color change. Since there is no hydrogen peroxide in their formulation, it is not possible to lighten the hair, but it is possible to darken the natural hair color by up to three shades.^14,25,32^ They penetrate superficially into the hair cortex and are fixed by weak polar interactions and van der Waals interactions, causing the color to remain longer than the temporary dyes (6 to 12 washes).^14,25^ Figure 1E illustrates the fixation of the semipermanent hair dyes inside hair strands.

Semipermanent dyes are derived from nitro compounds (mainly nitrobenzene), generally characterized by nitroanilines, nitrophenylenediamines, and nitroaminophenols.^25,32^ Most semipermanent dyes have low molecular weight and contain different auxochromic groups, which accentuate the color of the nitro group (chromophore group) present in the chemical structure of the dye.^32,35^ Some formulations mix about 10 to 12 different dyes to obtain the desired color.^32,35^ However, it is also possible to find in some semipermanent dye formulations some acidic and basic temporary dyes that contain −COOH or −SO_3_H groups in their structure, such as Acid Orange 7, Acid Violet 43, Basic Red 22, and Basic Blue 47.^7,32,34,35^ Figure 2A shows the chemical structure of some compounds used as semipermanent hair dyes.

**Figure 2:**
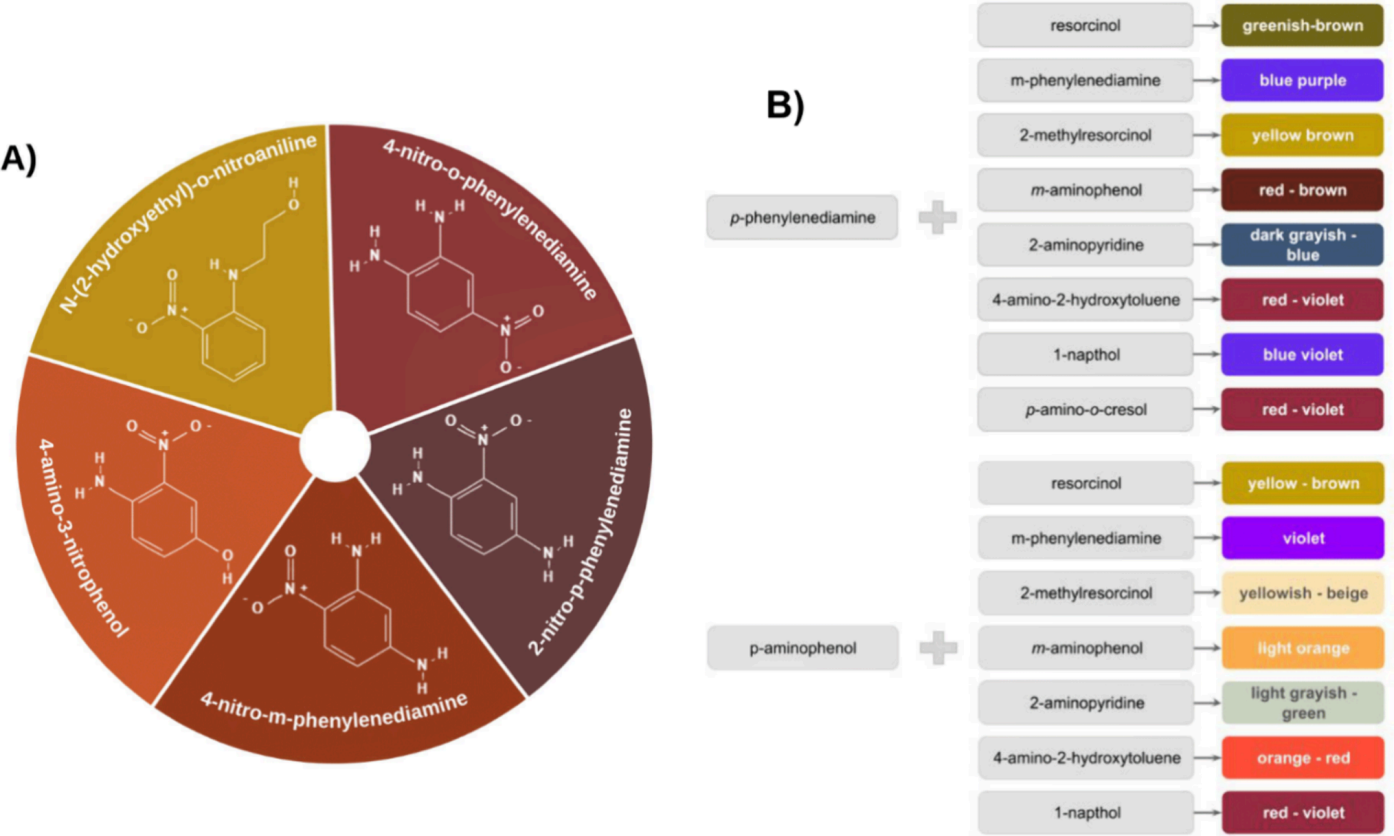
Dyes commonly used in semipermanent hair dye formulations (A) and colorations obtained with different combinations of precursors and couplers used in commercial permanent hair dye formulations (B).

### 2.3 Permanent Hair Dyes

Lastly, permanent dyes account for around 70–80% of the synthetic dyes applied on the world market, mainly due to their durability, versatility, and ease of application.^30^ They comprise a primary intermediate or precursor agent, a coupling agent or modifier, and an oxidizing agent.^2,7,9,18,25,28,29,33,36,37^ The final color shade depends on the composition, quantity, and nature of all of the products used. It also depends on the pH, reaction time, temperature, and the speed at which the components diffuse into the hair fiber.^2,7,9,18,25,28,29,33,36,37^ Thus, dyeing hair with permanent dyes is an art dictated by the chemical kinetics of a complex reaction and the diffusion mechanisms involved in the process.^7,18^ Figure 2B illustrates examples of the color obtained through the reaction between precursors and couplers commonly used in commercial hair dye formulations.

The main intermediate or precursor consists of aromatic amines, substituted at the *ortho* and *para* positions, containing amino and hydroxyl groups, such as PPD, PTD, and their derivatives. The concentration of these precursors in hair dyes depends on the desired shade, ranging from 0.05% for lighter colors to 1.5% for darker ones.^2,7,9,18,25,28,29,33,36,37^ The coupling agent or modifier consists of *meta*-substituted aromatic derivatives, including *m*-phenylenediamines, *m*-aminophenols, resorcinol (RSN), and naphthol, among others. These agents play a crucial role in defining the dye’s final color, functioning as electron-donating substances that react with the oxidized form of the primary intermediates in an approximate molar ratio of 1:1. Additional oxidative coupling reactions follow this process. In an alkaline medium, the oxidizing agent lightens melanin, the natural pigment responsible for hair color in the hair shaft, oxidizing the mixture’s precursor agent and other intermediate compounds and creating the desired color.^7,18,25,28,36^ While hydrogen peroxide is the most commonly used oxidizing agent, alternatives like urea peroxide, sodium percarbonate, and sodium perborate, often combined with ammonia, are less frequently employed.^7,9,28,29,36,37^ Lastly, alkalizing agents such as ammonia, monoethanolamine, and aminomethylpropanol are used, with ammonia being the most commonly utilized due to its efficiency in lightening the natural pigmentation of hair, enhancing the oxidation rate of precursors, and aiding in the swelling and opening of the cuticles, which improves the absorption of both dyes and the oxidizing agent.^2,9,25,33,36^

In general, the chemical compounds present in permanent synthetic hair dyes such as precursors, couplers, and additives, among others, are considered, in a way, the most reactive compounds in the cosmetics industry, which makes the formation of permanent color a complex process that sequentially involves the oxidation of the primary intermediate with several couplers.^7,9,18,33,37^ The permanent dyeing process involves mixing the precursor and the coupling agent with hydrogen peroxide in an alkaline medium (pH between 8 and 10). This mixture forms a cream that is applied directly to the hair, causing the precursors and coupling agents, together with the hydrogen peroxide, to diffuse into the hair fiber, forming a colored compound absorbed by the hair cortex after specific chemical reactions, providing persistent and stable color to the hair, which is the hair dye itself.^7,9,18,25,29,33^

A classic example of the formation of permanent dyes is the reaction between the precursor PPD and the coupler RSN in an alkaline medium (ammonium hydroxide) in the presence of hydrogen peroxide,^8,18,33,37^ presented in Figure 3. In the first stage of the reaction (route A, Figure 3), the PPD is oxidized first to a semiquinonediimine radical and then to quinone-diimine, which subsequently reacts with the nucleophilic coupler (RSN) to form the colorless leuco dye. However, the leuco dye is oxidized and converted to idoalinine dye, which has a red color, inside the hair shaft. The idoalinine dye reacts with a molecule of the primary intermediate quinone-diimine to form the green idoalinine dye, which continues to polymerize until it forms a large polymeric chain of polyindophenol. Polyindophenol is characterized by forming a brown precipitate, which also provides an identical color to hair.^8,18,33,37^ The presence of couplers in the composition of hair dyes has the function of guiding the reaction toward the formation of the dye of the desired color, and this reduces the formation of byproducts generated by the oxidation of amines exposed to light, oxygen dissolved in the environment, and oxidizing agents (e.g., hydrogen peroxide).^2,7,9,18,25,28,29,33,36,37^ As shown in route B in Figure 3, the autoxidation and polymerization of PPD can form the Bandrowski Base (BB), a highly toxic purple coloring byproduct.^7,18,37^

**Figure 3:**
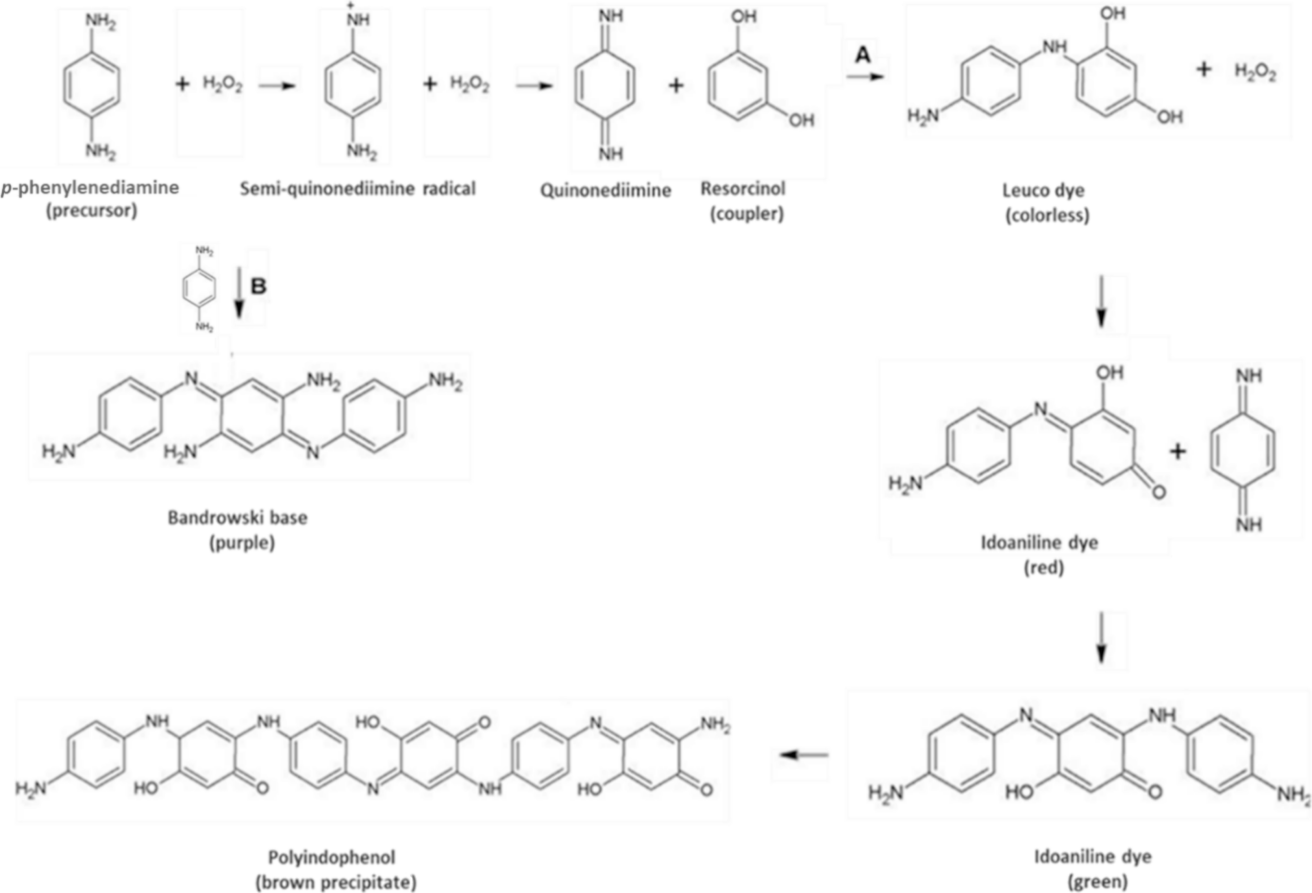
Chemical process of dyeing hair in the presence of hydrogen peroxide and ammonia with (A) the main products formed by the chemical reaction between PPD and RSN and (B) the byproducts produced through the oxidation of PPD.

Adapted with permission from ref 7. Copyright 2014, Sociedade Brasileira de Química.

The final shade achieved depends on the composition, quantity, and nature of all products utilized in the formulation. Additional factors influencing color development include pH, reaction time, temperature, and the diffusion rate of components into the hair fiber.^7,18^ Consequently, dyeing hair with permanent colorants represents a complex process governed by chemical kinetics and diffusion mechanisms operating within the hair structure. Following a standard dyeing procedure, hair contains various chemical compounds essential for color development, persistence, and stability, alongside a diverse range of dyes with distinct characteristics. Table S1 presents the spectroscopic properties of selected hair dyes that directly correlate with their coloration attributes and include relevant toxicological and ecotoxicological aspects associated with these compounds.^38–45^

## 3 Toxicity of Hair Dyes

It is known that during the dyeing process, a significant part of dye components is washed and released to the environment as salon wastewater been possible to cause different reactions.^47–57^ However, the primary concern associated with these dyes revolves around their disposal. When salon waste reaches its way into nearby water bodies, it carries many dyes and other substances present in hair dye formulations, which can harm water resources, soil fertility, aquatic organisms, and ultimately human health through the food chain.^30,58–60^ Consequently, the discharge of these dyes has an adverse aesthetic impact and introduces toxic chemical constituents into the environment.^58^

In a directive, the European Economic Community Cosmetics has established limit values for the maximum permitted concentration of various phenylenediamines in permanent hair dye compositions and henna dyes. These values are 6% for PPD and 10% for PTD, provided these products have excess couplers in their composition.^20^ Few studies have examined these compounds, their products, byproducts, wastewater from beauty salons, and their presence in surface water and drinking water. This lack of information is concerning, because these effluents often enter city sewage systems and can contaminate water reservoirs. Additionally, conventional water treatment methods are ineffective in handling this type of waste.^59–61^

### 3.1 Health Risks and Toxicological Mechanisms

As mentioned, hair dyes contain chemical agents that can trigger skin sensitization, potentially leading to allergic reactions such as contact dermatitis, particularly among frequent users like hairdressers and consumers.^7,18,19^ These substances can stimulate inflammatory immune cells and activate regulatory pathways, which may help explain why repeated use does not always result in visible allergic responses. Additionally, the possible cancer risks associated with hair dye components have been a long-standing concern in toxicology and epidemiology. This is mainly due to the presence of arylamines in oxidative dye formulations—a group of chemicals that includes known carcinogens such as benzidine, 4-aminobiphenyl, and 2-naphthylamine.^2^

The amines used as precursors can be easily oxidized when exposed, for example, to sunlight or oxygen dissolved in the environment,^20,62,63^ and can polymerize and form highly toxic byproducts, such as BB, which is produced by the oxidation and subsequent polymerization of PPD.^7,20,64^

The presence of couplers in the composition of hair dyes has the function of forming dye with the desired color, reducing the formation of byproducts from the precursors’ oxidation. However, even under the conditions determined by the manufacturer, not all the precursors in the formulation react with the coupler, so a certain amount of these byproducts is formed.^20^ Therefore, a study of the properties, the behavior of the reaction mechanism, and the products and byproducts that are generated from these amines are of great value and extremely necessary for understanding the hair dyeing process. Figure 4 shows the molecular structures of some toxic components present in hair dyes.

**Figure 4:**
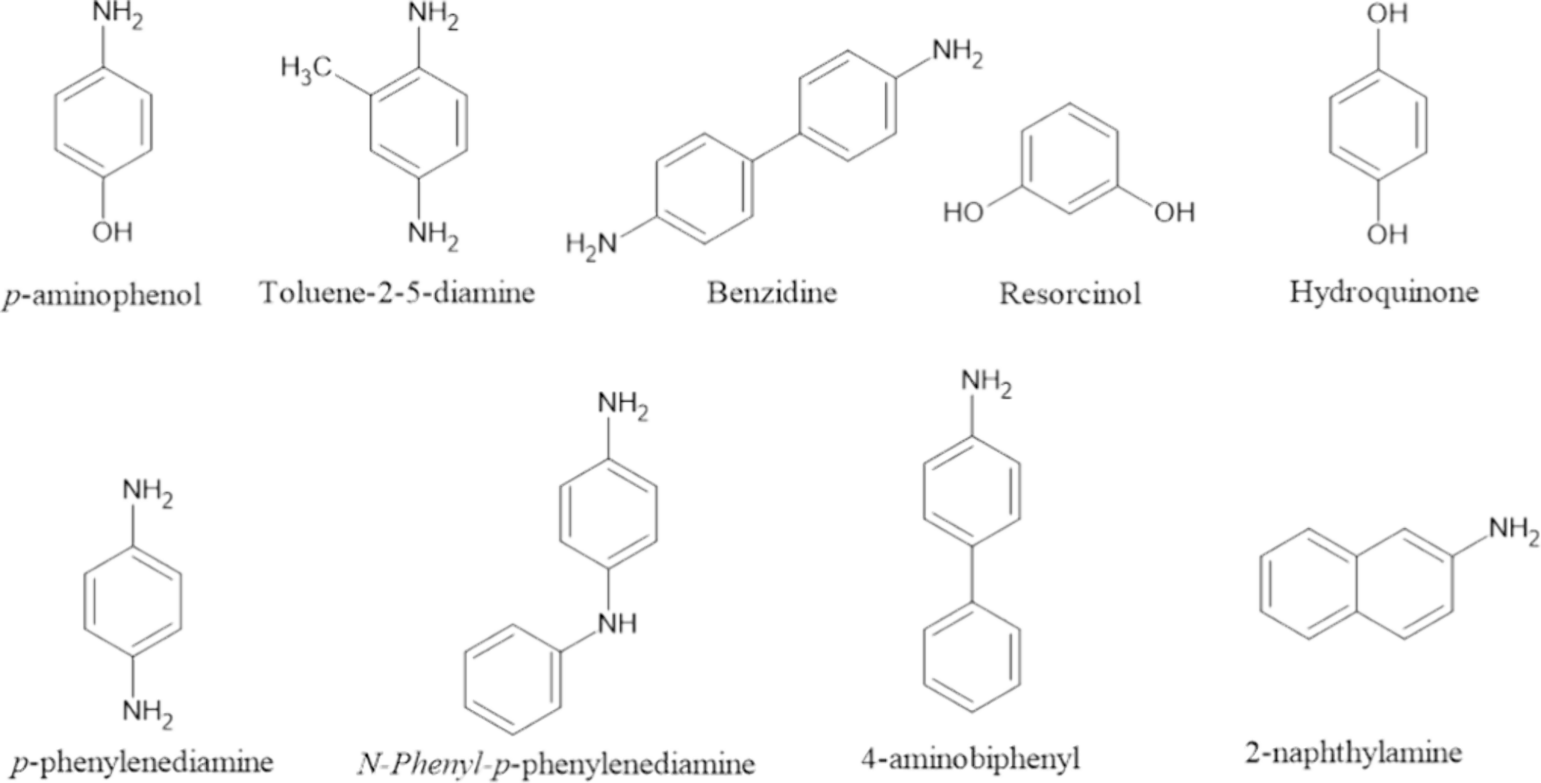
Molecular structure of some toxic components present in hair dyes.

Numerous epidemiological studies have raised concerns regarding an elevated risk of bladder cancer associated with hair dyeing, particularly among professionals, such as hairdressers and barbers, with occupational exposure to hair dyes. Some studies, including those by Gago-Dominguez et al.,^65^ Andrew et al.,^66^ and Harling et al.,^67^ have suggested a potential link between hair dye use and an increased risk of bladder cancer. Conversely, other research, such as that conducted by Baan et al.,^68^ Ros et al.,^69^ and Turati et al.,^70^ did not find supporting evidence for such an association. Considering the significant risk of urinary bladder cancer, the occupation of hairdressing has been classified as ″probably carcinogenic to humans″ (Group 2A) by the International Agency for Research on Cancer (IARC).^71^ Hair dyeing has also been investigated concerning other cancer types, including breast and hematological cancers, although, much like bladder cancer, the results remain inconclusive.^72–74^

When considering the potential pregnancy risks, research has identified links between pregnant women’s exposure to hair dyes and various health outcomes in their offspring. These outcomes include the development of leukemia up to the age of 2 years,^75^ neuroblastoma,^76^ and an increased likelihood of allergic rhinitis and asthma in offspring at 3 years.^77^ Another study has shown an association between prepregnancy hair dye use or irregular menstruation and abnormal birth weight. Notably, when these two factors coexist, the risk of low-birth-weight infants is further elevated.^78^ However, there is no consensus in the literature, and the results are conflicting.^75–78^

The American Pregnancy Association^79^ states that most research suggests that the chemicals in semipermanent and permanent hair dyes are generally not highly toxic and can be used safely during pregnancy. Moreover, only small quantities of these hair dyes are typically absorbed by the skin, making it unlikely for a significant amount to reach the developing fetus.^18,22,80^ Consequently, this minimal absorption is not considered a risk to the fetus. A similar principle applies to breastfeeding. Although there is a lack of specific data regarding women receiving hair treatments while breastfeeding, it is well established that only a tiny fraction of these chemicals enters the bloodstream. Therefore, the chances of these substances entering breast milk and potentially posing a risk to an infant are considered quite remote.^79^

As discussed previously, oxidative hair dye comprises essential components such as primary intermediates (phenylenediamines), couplers (*meta*-substituted aromatic derivatives), oxidants (hydrogen peroxide), and alkaline agents (ammonia).^2,7,9,18,25,28,29,33,36,37^ PPD and its derivatives are recognized as potent skin sensitizers, with positive patch test reactions to PPD frequently observed among patients with dermatitis.^81^ Also, hair dyes that contain PPD have raised health concerns due to their potential links to cancer and genetic mutations, as indicated by findings from both experimental research and clinical observations.^82^ Additionally, up to 84% of PPD is estimated to go unused during the hair dyeing process, eventually entering wastewater, thereby becoming an emerging environmental concern.^26^

However, the main adverse effect of PPD doses in humans and other higher mammals is angioneurotic; that is, there is the formation of edema in the lungs that causes acute respiratory disorders. Furthermore, PPD can cause gastritis, renal failure, dizziness, tremors, convulsions, coma, and rhabdomyolysis, which is the necrosis of skeletal muscle, resulting in acute renal failure, as well as atrophy of the optic nerve.^18,19^

*N*-Phenyl-*p*-phenylenediamine is a commonly encountered aromatic amine found in hair dyes, and it has been linked to various adverse effects including skin sensitization, a finding supported by the EU Scientific Committee on Consumer Products (SCCP). In hair dye formulations, hydroquinone serves multiple purposes as an antioxidant, fragrance, reducing agent, and polymerization inhibitor. However, it can induce nephrotoxicity, cytotoxicity, skin irritation, sensitization, depigmentation, and mutagenicity. Toluene-2,5-diamine and toluene-3,4-diamine, two isomeric compounds employed in hair dyes, have been associated with unfavorable effects, notably marked skin sensitization and reproductive toxicity.^83^ RSN, a widely used component in hair dyes and cosmetics, disrupted thyroid hormone synthesis and caused goitrogenic effects after administration to rodents at high doses (>520 mg/kg/day) over 2 years.^84^

The chemical compounds found in permanent synthetic hair dyes, such as precursors, couplers, additives, and others, are considered, in a way, the most reactive compounds in the cosmetics industry, which makes the hair dyeing process quite complex.^7,9,28^ This means that the toxicological potential of these dyes is responsible not only for the pure compounds used in the compositions of these products but also for the secondary products and byproducts generated by the oxidation of precursors and couplers during the hair dyeing process. A classic example is the formation of BB by the oxidation of PPD. In addition to being an undesirable byproduct in the hair dyeing process, the formation of BB has raised significant concern since some authors indicate that BB is a strong sensitizer, a potent allergen, and acts in hair loss, in addition to being highly genotoxic, mutagenic, and carcinogenic.^85–88^ Figure 5 presents the possible risks of dye hair exposure in the human body.

**Figure 5:**
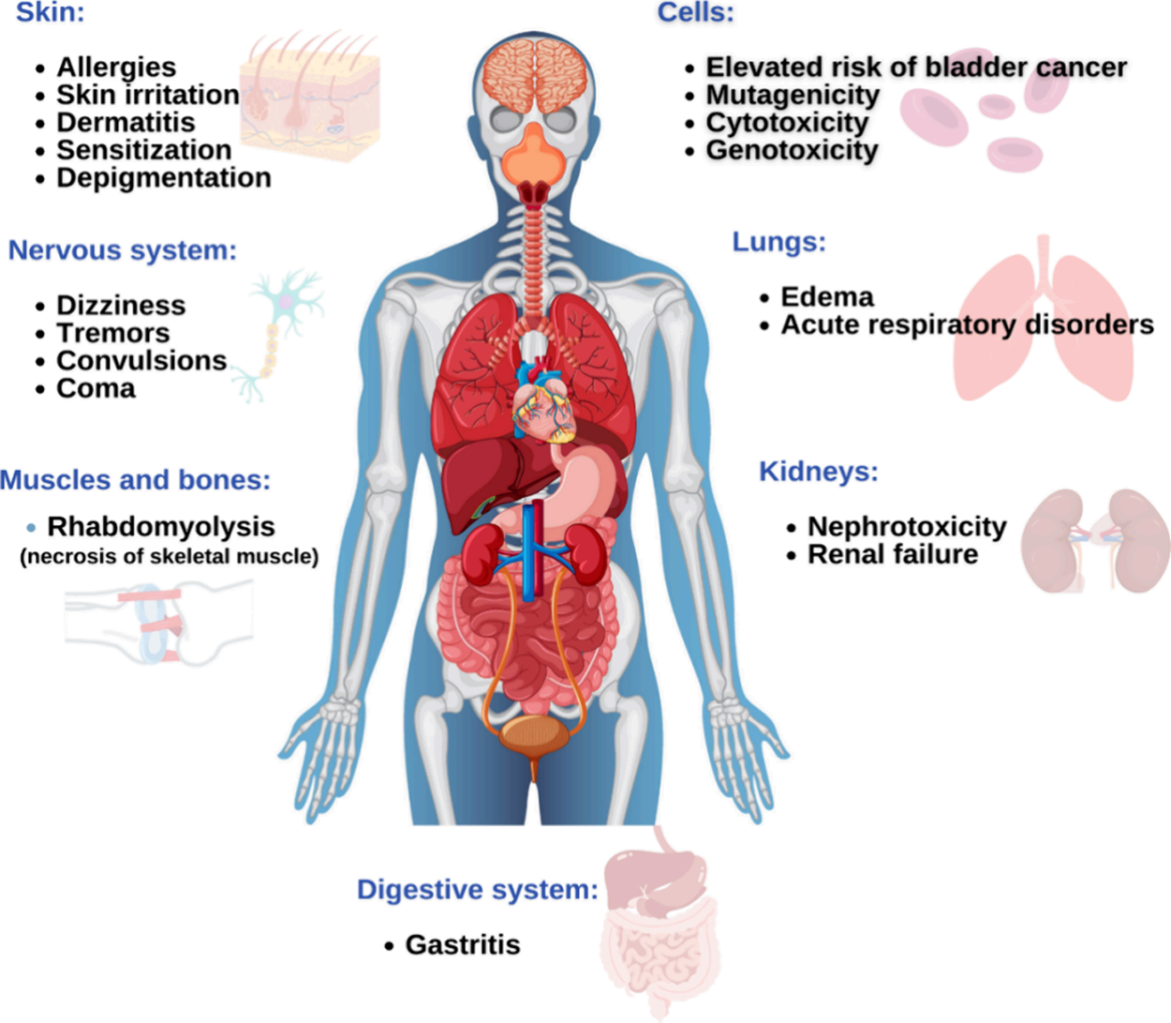
Possible health risks for humans in different body areas, such as muscles and bones, skin, lungs, kidneys, digestive system, nervous system, and cells, caused by prolonged exposure to hair dye.

The hair dye’s toxicity mechanism has been little investigated, and the data is still conflicting. It is known that the toxicity of hair dyes is associated with their constituents. Aromatic amines such as PPD constitute the primary compounds used as precursors in permanent hair dyes. Studies have extensively demonstrated the toxicological properties of PPD, including its role in inducing apoptosis via increased reactive oxygen species production.^89^ During the hair dyeing process, PPD can penetrate the skin or be absorbed through the respiratory system, where it undergoes biotransformation into *N*-monoacetyl-*p*-phenylenediamine (MAPPD) and *N*,*N*′-diacetyl-*p*-phenylenediamine (DAPPD), as shown in Figure 6A.^83,90^ The metabolite profile is dose-dependent: concentrations between 250 and 1000 μM favor MAPPD, whereas lower concentrations (<250 μM) predominantly result in DAPPD formation.

**Figure 6:**
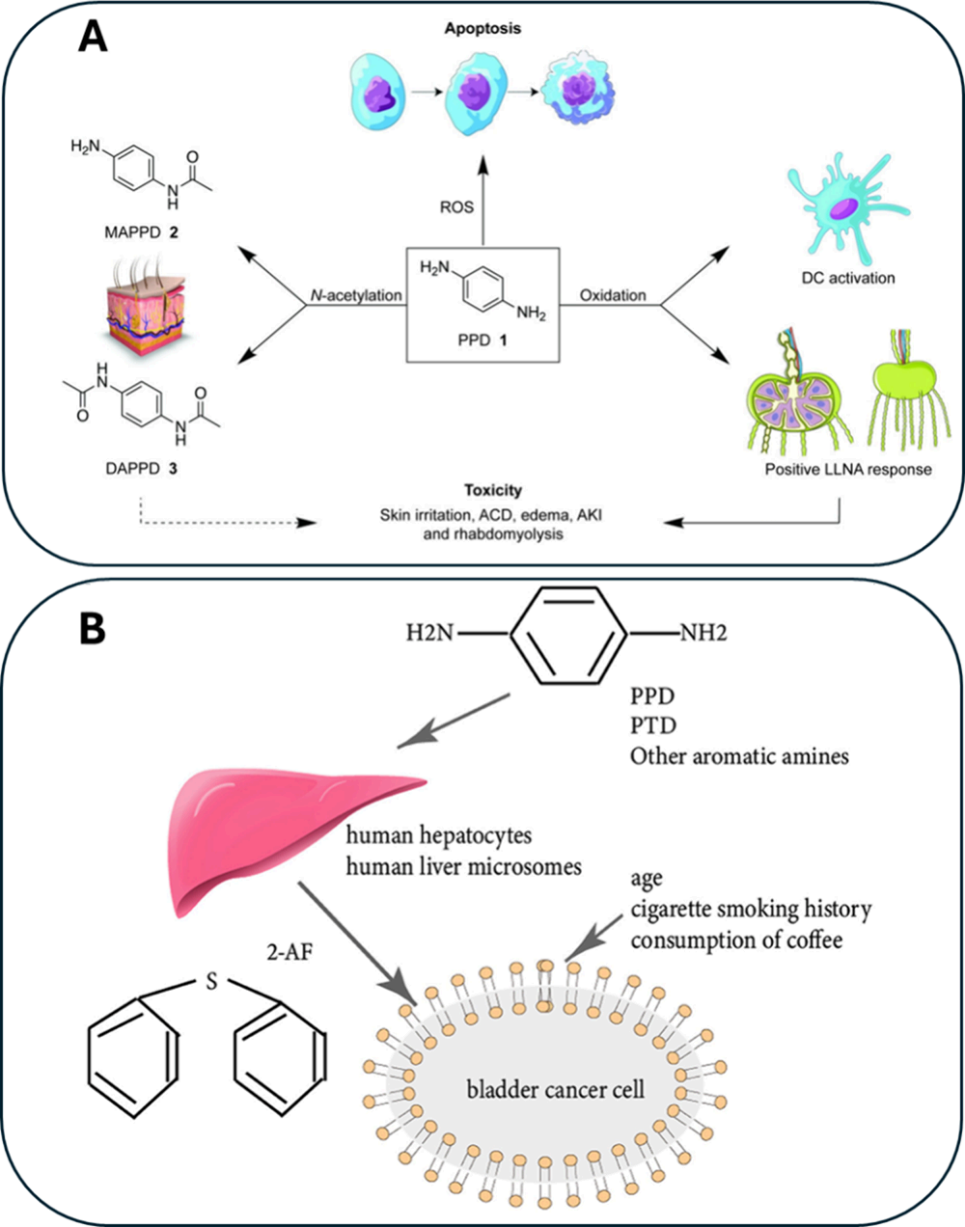
Mechanism of toxicity induced by PPD (A), and possible carcinogenesis mechanism of chemicals in hair products (B). Reprinted with permission from He, L.; Michailidou, F.; Gahlon, H. L.; Zeng, W. Hair Dye Ingredients and Potential Health Risks from Exposure to Hair Dyeing.^83^ Chemical Research in Toxicology. American Chemical Society June 20, 2022, pp 901–915. Copyright 2022, American Chemical Society.

Additionally, PPD oxidation produces sensitizing agents that activate dendritic cells and elicit immune responses, as shown in *in vitro* and *in vivo* models.^86^ In contrast, MAPPD and DAPPD do not provoke such sensitization. The biotransformation of PPD into MAPPD/DAPPD and the oxidative formation of sensitizers represent two competing metabolic pathways. Higher concentrations of PPD promote sensitizer formation, exacerbating PPD-related toxicities.^83^ PPD metabolism in the skin mainly involves oxidation and acetylation pathways. Haptens, metabolites that trigger sensitization reactions, are formed through oxidative processes. Less than 1% of free PPD undergoes acetylation reactions in the epidermis without activating T or dendritic cells in sensitized individuals; therefore, the enzymes responsible for acetylation reactions become saturated, which increases oxidation reactions. This results in prolonged skin exposure to high doses of sensitizing metabolites and contamination of the adjacent skin.^92^ In addition to PPD, substances such as PTD, PAP, *m*-aminophenol, RSN, monoethanolamine, ammonium persulfates, ammonium thioglycolates, glyceryl thioglycolates, and sodium metabisulfite can lead to contact dermatitis in users of hair colorants.^93^ Moreover, PPD is believed to induce rhabdomyolysis through calcium release and leakage of calcium ions from the smooth endoplasmic reticulum, which causes intense muscle contraction and irreversible changes in the muscle structure. Rhabdomyolysis is the leading cause of acute renal failure, probably due to the combination of rhabdomyolysis, hypovolemia, and the toxic effects of PPD on the kidneys.^94^

Another toxic effect associated with PPD is respiratory syndrome, which is represented by asphyxia and respiratory failure secondary to inflammatory edema. The mechanism is believed to be due to precipitous inflammatory edema of the cricopharyngeal and laryngeal structures. Histologic changes of acute tubular necrosis have been described in PPD poisoning.^94^ Some studies on the mechanism of carcinogenic action of hair products have shown that the formation of an *N*-hydroxylamine by *N*-oxidation in the human liver is crucial in the development of bladder cancer. PPD, an important arylamine present in many oxidative hair products, is converted to 2-aminofluorene (2-AF), a bladder carcinogen (Figure 6B). Exposure to 2-AF associated with other risk factors such as age, cigarette, and coffee consumption, has great potential to induce bladder cancer.^91^

Other aromatic amines, such as PTD and PAP, precursors detected in permanent hair dyes, are also associated with the development of bladder cancer.^91^ Souza et al. showed that the compounds generated by the oxidation reaction involving these aromatic amines presented mutagenic properties in Salmonella assay, illustrating the cancer potential of commercial hair dyes by point mutations.^30^

Some studies show that the presence of aromatic amines in hair products may be associated with the incidence of breast cancer in women exposed during adolescence since breast tissue is highly vulnerable to these components in adolescence.^95^ Ambrosone et al. found 4-aminobiphenyl-DNA adducts associated with hair dye use in breast milk epithelial cells, indicating that these compounds circulate in the body and reach DNA-forming adducts, which is a potential carcinogenic mechanism.^96^

RSN not only is a dermal sensitizer but also exhibits properties that disrupt thyroid function. This compound is a known inhibitor of thyroperoxidase (TPO), an enzyme essential for synthesizing thyroid hormone.^97^ TPO catalyzes several reactions, including iodination of tyrosyl residues in thyroglobulin and subsequent oxidative coupling to yield thyroxine (T4) and triiodothyronine (T3).^98^ Inhibition of such TPO functions may reduce blood TH levels, resulting in thyroid hyperplasia and developmental abnormalities or neurological dysfunction.^99^ Human data show severe clinical hypothyroidism with associated goiter.^100^

While there exists conflicting evidence regarding the toxic effects of hair dyes following occupational exposure or personal use, there remains a notable gap in our understanding regarding their impact on the environment and potential health risks for individuals exposed to low concentrations of these substances in water. Given the rising number of users and the expanding economic influence of the hair dye industry, the practice of hair dyeing has emerged as a pressing public health concern, demanding an urgent assessment of the toxicity and carcinogenic potential associated with hair dyes, as emphasized by He et al.^83^ Furthermore, it is of utmost importance to advance techniques for detecting and treating these compounds in environmental samples.

### 3.2 Emerging Alternatives and Future Directions

Recently, a great deal of effort has been devoted to finding dyes adaptable to more diverse permanent dyeing, and in this context, many of the mechanisms involved in dyeing textile products have also been studied. It is important to note that the dyeing of cotton, wool, and leather, for example, are processes based on the interaction between dye molecules and groups, −OH, −NH_2_ present in cellulose, amino acids, and proteins present in these materials, which are also present in hair.^7^ Several studies have investigated the in situ formation of insoluble azo dyes within the hair structure, often involving reactions between diazonium salts and coupling agents.^101^ A related approach involves adapting VAT-type dyes—commonly used for dyeing cellulose fibers—for hair coloring. Although these dyes are typically water-insoluble, they can be temporarily converted into a soluble leuco form under alkaline conditions. Once applied to the hair, they revert to their original, less soluble state through oxidation, either by air exposure or with the help of an oxidizing agent.^7^

In addition, literature has described the application of reactive dyes, which form stable covalent bonds with substrates such as cotton or silk that contain functional groups such as hydroxyl, amino, or thiol groups. Similar principles have been tested for hair, where the reactive dye groups are designed to bond with amino acids in the hair protein structure.^102,103^ Efforts have also been directed toward artificially generating melanin within the hair fiber using biochemical pathways that emulate natural pigment formation.

These techniques are technically challenging due to the complex interplay of factors such as pigment type, particle size, density, and distribution within the hair shaft, all of which affect the final appearance. Among the experimental approaches, the enzymatic or oxidative synthesis of melanin analogs like eumelanin and pheomelanin has shown the most encouraging outcomes.^7,103^ However, despite their potential, these methods remain under development and are not yet viable for widespread commercial application.

## 4 Advancements in the Detection of Hair Dyes

The hair dye industry has experienced remarkable growth in recent years, driven by increased popularization, technological advancements, and the development of new formulations offering diverse colors, enhanced fixing power, improved durability, better safety profiles, and greater accessibility through competitive pricing. These factors have transformed the cosmetics market into an increasingly profitable sector.^7,28,60,104^

This expanding consumption necessitates stringent manufacturing controls to ensure standardized, high-quality products with optimal physicochemical characteristics while minimizing potential risks and side effects for users.^105^ This requirement is particularly crucial given the documented toxicological concerns. Numerous studies have demonstrated that hair dyes and their components (precursors and couplers) exhibit significant toxicity, mutagenicity, genotoxicity, and carcinogenicity. Users may experience adverse reactions, including allergies, dermatitis, skin eruptions, headaches, seizures, bronchial asthma, and damage to vital organs such as the blood, stomach, lungs, liver, kidneys, and brain. Additionally, these substances pose substantial environmental risks, particularly to aquatic ecosystems.^7,28,30,59,60,105^ Growing environmental concerns have prompted increased research on detecting dye traces, precursors, and couplers in aquatic environments.^59,60,106–113^

Developing reliable analytical methods for detecting and quantifying hair dyes and their derivatives in various matrices (cosmetics, water, biological fluids) is essential for environmental monitoring and public health protection.^7,28,60,104,105^ Several analytical techniques have been employed, including paper chromatography, thin layer chromatography (TLC), gas chromatography (GC), high-performance liquid chromatography (HPLC) coupled to various detectors, capillary electrophoresis (CE), UV–vis spectrophotometry, and electroanalytical methods (Figure 7).

**Figure 7:**
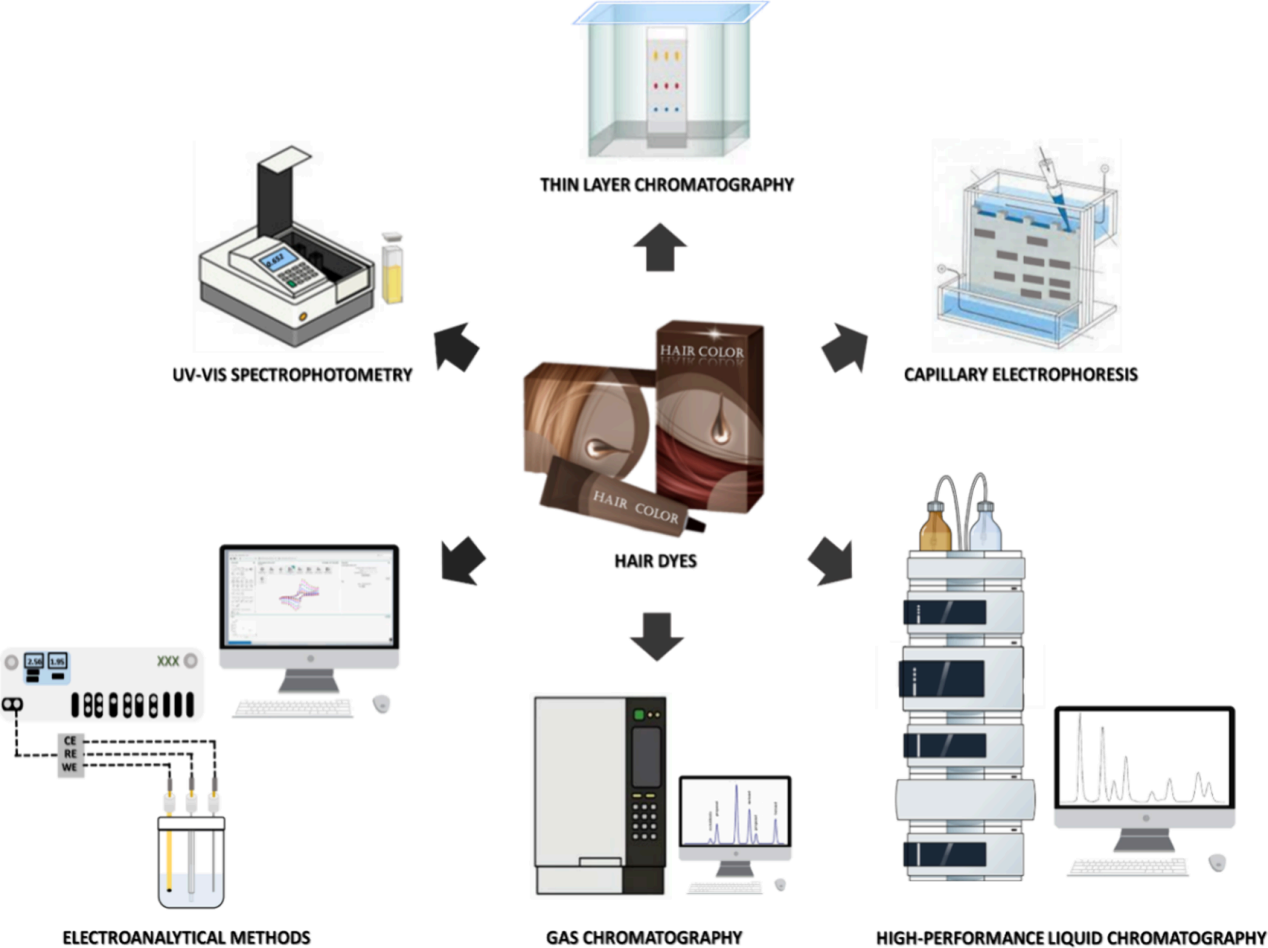
Analytical techniques such as UV–vis spectrophotometry, capillary electrophoresis, electroanalytical sensors; thin layer, gas, and liquid chromatography, which can be applied to detecting, determining, and quantifying hair dyes and their derivatives.

Paper chromatography, one of the earliest techniques, provided qualitative analysis of hair dye mixtures.^7^ A notable study by Smith and McKeown^114^ in the 1960s enabled the separation of 29 compounds used as precursors and couplers, such as aryldiamines, aminophenols, and polyhydric phenols. However, limitations in sensitivity and selectivity have rendered this technique largely obsolete.^7^ TLC emerged as an improved option for separation and identification, offering better selectivity and sensitivity at a moderate cost.^30,33,59,60^ Shah et al.^33^ developed a methodology to separate and identify eight compounds from the reaction between PPD and RSN, respectively, a precursor and a coupler widely used in commercial permanent hair dye formulations. Souza et al. also used the same technique to separate and identify products resulting from the oxidation reaction of the PTD precursor,^60^ the PAP coupler,^59^ and the reaction between them.^30^ Although TLC has better selectivity and resolution compared to PC, with the use of different stationary phases, it has also fallen out of favor after the emergence of the chromatographic techniques, such as GC and HPLC techniques, which have superior selectivity, separation, resolution, and sensitivity compared to TLC.^9^

GC offers superior separation capacity, resolution, and selectivity among chromatographic techniques.^9^ Using these advantageous characteristics, Schmidt et al.^115^ developed a GC method by derivatization through the reaction with iodine to determine 56 aromatic amines used in permanent hair dye formulations. The methodology proved quite sensitive, obtaining detection limits between 0.5 and 8.0 μg L^–1^. Despite this ability to separate several compounds simultaneously, GC has disadvantages such as meticulous sample preparation involving cleanup, extraction using volatile organic solvents, headspace, and derivatization reactions.^116^ These steps can make the proposed methodology expensive, with the possibility of increased errors and decreased analytical frequency due to the many sample preparation steps, in addition to the use of organic solvents that are relatively toxic to health and the environment, as well as the need for highly trained personnel to use the equipment.^9^

HPLC is the most widely adopted technique, offering excellent selectivity, sensitivity, and precision with a simpler sample preparation. When operated under optimal conditions of the mobile phase, stationary phase, and various detectors, the HPLC technique provides exceptional selectivity, sensitivity, and precision. Unlike GC, HPLC often requires minimal sample preparation, such as simple filtration or preconcentration.^59,60,105,117^ Scarpi et al.^118^ developed a methodology using HPLC coupled to a diode array detector (DAD) to determine 10 temporary hair dyes in commercial formulations. The proposed methodology proved highly attractive as it does not require a sample extraction procedure, with relatively low detection limits (1.0–5.0 μg mL^–1^), good precision, recovery, and analytical frequency.

Capillary electrophoresis has also been used to determine hair dyes and has proven to be an interesting tool due to its characteristics, such as high selectivity and excellent resolution.^7,9^ Compared with HPLC, capillary electrophoresis consumes fewer organic solvents but has lower sensitivity. Masukawa^119^ developed an analytical methodology to determine four temporary dyes (Basic Red 76, Basic Brown 16, Basic Yellow 57, Basic Brown 17, and Basic Blue 99) in hair care products using capillary electrophoresis. Under optimized acetic acid/ammonium acetate conditions containing methanol, relatively low detection limits were achieved on the order of 0.7–4.5 μg mL^–1^.

The necessity of fast monitoring methodologies with low environmental impact and minimum sample preparation for analysis of hair dye components drove the use of electroanalytical methods. The development of chemically modified electrodes, which can allow the identification and quantification of different analytes, simultaneously or not, quickly, specifically, and precisely at low concentrations and in complex matrices, are highly attractive alternatives due to their high robustness, selectivity, accuracy, precision, and sensitivity.^60,104,105,120^ The infinite possibilities of modifying electrodes allow the development of the most varied sensors to meet the demand in the most diverse areas, where both industry and environmental and health inspection bodies need sensors for quantitative or differential analysis of countless products and industrial residues, such as dyes and aromatic amines used in permanent hair dyes.^60,104,105,120,121^

Along these lines, Hudari et al.^104^ simultaneously determined the PPD precursor and the RSN coupler in samples of commercial formulations of hair dyes and tap water, using a simple, economical, selective, and highly sensitive voltammetric sensor consisting of a glassy carbon nanotubes electrode (GCE) coated with chitosan-modified multiwalled carbon nanotube composites (MWNTs–CHT/GCE). Modifying the glassy carbon electrode in the proposed methodology increased the current density by 10% for PPD and 70% for RSN compared to that of the unmodified electrode. The calibration curve showed linearity between 0.55 and 21.2 mg L^–1^ with detection limits of 0.79 and 0.58 mg L^–1^ for PPD and RSN, respectively, and recovery in samples at around 97%.

Although slightly less used and with some disadvantages compared to chromatographic techniques in terms of selectivity and sensitivity and, as most of them require derivatization and complexation reactions, the UV–vis spectrophotometry technique is also used to determine some compounds used in hair dye formulations. Zatar et al.^122^ developed a UV–vis spectrophotometry methodology for the determination of four aromatic amines (1,4-phenylenediamine, 2,4-diaminotoluene, 8-aminoquinoline, and 2-amino-3-hydroxypyridine), based on the reaction between the amine and the colorless Fe­(III)–ferrozine complex. In this reaction, the amine in the medium reduces Fe­(III) to Fe­(II), forming a violet complex with ferrozine.

Moreover, the literature offers a multitude of analytical methodologies, using the most diverse techniques for determining hair dyes and their derivatives. Table S2 provides details such as detection and quantification limits, percentage of recovery, and linear range related to different analytical techniques published around determining and quantifying analytes present in hair dyes and their derivatives in different matrices such as water, wastewater, urine, and blood.^123–131^

All the methods developed, together with all the techniques applied and exemplified (Table S2), provide attractive, reliable, and efficient alternatives for detecting, determining, quantifying, and monitoring hair dyes and their derivatives in several samples. The choice of the proper technique will rely on the situation and factors such as complexity, the concentration of the analyte, and interferents present in the sample, making one technique a more appropriate tool than another for the analysis. Given this, there must be a prior assessment of the analyst’s objective and what information they want to obtain when carrying out the analysis, in addition to making an initial characterization of the sample to choose which technique is most suitable for the intended purpose, considering its characteristics and its positive and negative points.

Taking into account the analytical techniques available in the literature and pointed out in this review, including paper chromatography, TLC, UV–vis spectrophotometry, CE, GC, and HPLC coupled to different detectors and electroanalytical methods based on the use of modified electrodes, HPLC is the most used and considered one of the most efficient ones for the determination of hair dyes and their ingredients and derivatives, due to their high specificity, resolution, separation capacity, sensitivity, and speed. On the other hand, it is a technique that requires using relatively expensive equipment (liquid chromatograph) operated by highly trained technicians, generating considerable wastewater from a liquid mobile phase. Furthermore, many methodologies that employ this technique involve using C18 reversed-phase chromatographic columns, which have poor retention capacity for this type of compound and do not offer reliable quantification.^9^ To address this challenge, specific methodologies suggest incorporating reagent ions (ionic liquids) into the mobile phase. This approach facilitates the formation of complex ion pairs with analytes, thereby enhancing retention capacity and improving the separation of analytes and the resolution of chromatographic peaks.^9,132^ However, suppression effects and low volatility may restrict the use of ionic liquids. Another option would be high-polarity stationary phases to provide a greater retention of the analytes.^9^ Another option that should be highlighted and that can also be employed to improve the detection capability of other techniques is the possibility of cleaning up the sample and preconcentrating the analytes via liquid–liquid and solid–liquid extraction. However, that option makes the analysis even more complex and laborious.^9^

Although GC is the technique with the greatest selectivity, separation power, and resolution among all those mentioned, it is still little used for analyzing dyes due to the low volatility of these compounds and the need for parallel derivatization reactions.^116^ Furthermore, similar to HPLC, there is a need to use relatively expensive equipment (gas chromatograph) and trained operators for the equipment. In most cases, extractions are also needed for these analyses, making GC more laborious and a less attractive alternative to HPLC and electroanalytical methods, for example.^133–160^

To overcome all the problems associated with HPLC and GC, electroanalytical methodologies based on the use of modified electrodes present a very interesting alternative since such methodologies are highly sensitive, fast, and cheap and require almost no or no treatment sample compared to other techniques. However, in some cases, mainly in the analysis of dyes and their derivatives, electroanalytical techniques present difficulties in terms of selectivity, as many of the molecules of these compounds are very similar and have very similar electroactive groups, oxidizing and reducing at very close potentials, thus hindering their determination.^105,121^

Therefore, it is vital to know and understand the several analysis methodologies available in the literature so that the analyst can choose which methodology best suits their needs according to their characteristics. More robust, sensitive, and reliable determinations can be made from this.

## 5 Progress in the Treatment of Wastewater Contaminated by Hair Dyes

In order to mitigate the environmental impact caused by the disposal of hair dyes, significant advancements have been made in the development of treatment methods. Removing hair dyes from wastewater has become increasingly critical due to their potential environmental and health hazards.^7,28,30,59,60,105^ This section delves into various strategies to treat hair dyes, highlighting the progression from adsorption to more advanced techniques such as biodegradation, photocatalysis (PC), photoelectrocatalysis (PEC), and catalytic ozonation.

Researchers have explored diverse adsorption-based approaches in the quest for advanced treatment methods for hair dye wastewater, each offering unique insights and advantages. The water removal of hair dyes using adsorption employing a powder of oak cupules (COZ) coated with ZnO was investigated by Al-Ma’abreh et al.^161^ They examined the adsorption characteristics of three hair dyes: Arianor madder red (AR), Arianor straw yellow (AY), and Arianor ebony on oaks (AE). COZ exhibited an impressive adsorption capacity, with 55.5 mg g^–1^ for AR, 52.6 mg g^–1^ for AY, and 135.1 mg g^–1^ for AE. This adsorbent displayed excellent reusability after five regeneration cycles.

Using simple, abundant, and nontoxic materials as adsorbents appears to be an environmentally friendly approach for that goal, including some of the possibilities of agricultural waste. Durian shell was investigated as an eco-friendly adsorbent for removing Basic Brown 16 (BB16) from aqueous solutions.^162^ Under optimized conditions (pH 8, 30 min contact time, 1.0 g L^–1^ durian shell dosage, and 15 mg L^–1^ BB16 concentration), the process achieved a notable 77.6% BB16 removal and 80.6% reduction in chemical oxygen demand (COD). Another example is the carbonization of bagasse, which generates iron–carbon hybrid magnetic nanosheets with a layered structure of mesoporous adsorbents exhibiting a high specific surface area (∼462 m^2^ g^–1^).^163^ This adsorber can remove various organic dyes and 4-nitrophenol from aqueous solutions. In this case, it was applied to remove a commercial hair dye that reached 92.6% in 20 min, demonstrating the applicability of this approach in wastewater treatment. Additionally, these adsorbents maintain structural stability and are easily detachable by using an external magnetic field, making them highly recyclable, even after five cycles.

While adsorption techniques have efficiently removed hair dyes from aqueous solutions, there is a necessity for other approaches for the detoxification of water contaminated by these dyes. In contrast to the adsorption technique, which captures hair dyes physically, the detoxification method employs a biodegradation process to break down the substances. Bioremediation involves using living organisms, such as microorganisms or plants, to remove or neutralize environmental pollutants. In the context of hair dyes, bioremediation offers a sustainable and efficient alternative to mitigate the environmental impacts of these compounds. A notable contribution to this field is the study conducted by Maiti et al., which represents a key effort in addressing the biodegradation of hair dye pollutants in wastewater.^58^ This research used sugar cane bagasse powder (SBP) as a nutrient source and a surface for bacterial cultivation. Through 16S rDNA sequencing, the bacterial isolate was identified as *Enterobacter cloacae*, labeled DDB I. Remarkably, 1 mg mL^–1^ of dye was successfully decolorized within 18 h of treatment with DDB I in a minimal medium supplemented with 30 mg mL^–1^ of SBP.^58^ Although there is an extensive body of literature on the bioremediation of industrial dyes, such as azo and triarylmethane dyes commonly found in textile effluents, studies focusing on the bioremediation of hair-dye-specific compounds, like PPD, remain scarce. PPD is a toxic aromatic compound used extensively in hair dye formulations and various industries.^164^ Bacterial strains are harnessed to detoxify PPD, offering an eco-friendly, cost-effective solution. Using the dye-degrading bacteria DDB I (KX881076), Maiti and colleagues found a significant decrease in oxidation (color formation) and evidence that PPD detoxification leads to the creation of less harmful compounds. The study found that biotransformed PPD demonstrated 100% detoxification, while PPD treated with hydrogen peroxide (H_2_O_2_) showed 77.0% detoxification of 0.2 mg mL^–1^ PPD at 30 °C and pH 5 for 12 h. These results signify the importance of controlling the oxidative environment, as the presence of hydrogen peroxide increased the toxicity of PPD.^164^

While adsorption-based methods efficiently capture and remove hair dyes from wastewater, detoxification processes are instrumental in rendering harmful compounds less toxic. These two strategies represent essential steps toward environmentally responsible wastewater treatment. However, it is worth noting that even after effective adsorption and detoxification, trace amounts of persistent and recalcitrant organic pollutants may remain in solution. Advanced oxidation processes (AOPs) effectively degrade persistent pollutants, ensuring top water quality and safety.^165^ By harnessing the power of AOPs, it becomes possible to tackle those elusive compounds that conventional methods struggle to eliminate, further contributing to the understanding and sustainable management of pollutants in our wastewater.

Photocatalysis (PC) is the simplest AOP based on a semiconductor excited by light of appropriate energy to generate hydroxyl radicals for organic degradation.^166^ Many semiconductors can be applied for that. Zinc oxide nanoparticles exhibit impressive efficiency in decolorizing a typical hair dye component, the Basic Red 51 (BR51), in hair dye greywater by PC. The approach leads to the decolorization of 72.2% of BR51 and the removal of 82.7% of COD.^166^

It is possible to assist the PC technique with an electrochemical potential to improve the degradation efficiency significantly, naming the technique photoelectrocatalysis, PEC.^167,168^ Several different semiconductor electrodes were already employed in the degradation of hair dyes, such as W/WO_3_ thin film,^46,169^ W/WO_3_/TiO_2_ bicomposites,^170^ and TiO_2_ nanotube photoanodes (TNT) and boron-doped TNT (B-TNT).^171^ The degradation of the BR 51 dye was also investigated under PEC oxidation using W/WO_3_ thin film under visible radiation, and complete decolorization was achieved after 60 min and 63% mineralization.^169^ This photocatalyst also achieved complete color removal and up to 59 and 44% mineralization of Basic Brown 16 and Basic Blue 99, respectively.^46^ TNT and B-TNT photoanodes were employed in the photo­(electro)­catalytic degradation of 100 mg L^–1^ Acid Yellow 1 hair dye (AY1), reaching 100% decolorization and up to 95% TOC removal. The extended absorption capabilities of B-TNT make it a suitable catalyst under visible light.

Another approach of AOP was based on a porous copper fiber sintered sheet loaded with Cu/Zn/Al/Zr catalysts for catalytic ozonation, leading to the effective degradation of Basic Yellow 87 (BY87).^172^ Compared to the ozonation process alone, this catalyst chip markedly improved degradation efficiency, demonstrating twice the effectiveness in removing COD and five times greater efficiency in removing TOC. Batch experiments confirmed the ability of porous copper fiber sintered sheet to sustain high removal rates, achieving approximately 99% for BY87, 60% for COD, and 30% for TOC. These outcomes remained consistent across a wide range of temperatures, BY87 concentrations, and ozone-to-BY87 molar ratios, all within a 4 h reaction period.

The first report of the combination of photoelectrocatalysis and ozonation was made by Bessegato and collaborators, where AY1 was the model compound.^173^ The researchers used an annular bubble reactor and optimized the electrochemical potential, ozone flow, and lamp emission (UV–B or UV–C). The decolorization rate constant (*k*) was 2.5 times higher for O_3_/PEC than for O_3_ and 1.9 times higher than for O_3_/PC, with the investigation of the AY1 degradation pathway by LC-MS/MS. The energy consumption (electrical energy per order) was also estimated, and it was found that the consumption of O_3_/PEC is about six times lower than that of O_3_. This innovative approach overcomes the challenges of PEC in conditions of reduced transparency in concentrated effluents, leading to faster decolorization and higher mineralization.

One of the biggest bottlenecks in recent publications in the field of AOPs is proving efficiency when using real effluents, which are complex and challenging. Grčić and colleagues assessed household greywater treatment after hair dyeing.^174^ They employed a solar PC using TiO_2_-coated textile fibers, followed by flocculation with dissolved chitosan. The treatment significantly reduced the organic content (83%), chemical oxygen demand (89%), and toxicity of YTT (EC_50_ (%)) (100%). The results showed a promising method for treating effluents laden with hair dye residues.

Another notable study focused on treating real hair dye effluent was performed by Bessegato et al.^61^ using different combinations of advanced oxidation processes. The study evaluated the effectiveness of O_3_, O_3_/UV–C, O_3_/PEC, O_3_/UV/H_2_O_2_, and O_3_/PEC/H_2_O_2_ in removing hair dyeing contaminants. Combining O_3_/PEC/H_2_O_2_ has emerged as a particularly effective method for treating hair dye wastewater. This combination has shown low electric energy per order and a high mineralization rate capable of entirely degrading harmful compounds such as PPD, RSN, and BB in less than 5 min. Furthermore, this combination has successfully eliminated unidentified compounds appearing as peaks in chromatograms without the formation of new degradation products. These findings are significant as they provide a crucial step toward developing an efficient and simple method for treating complex hair dye wastewater.

As can be observed, the diversity of approaches presented underscores the ongoing evolution in the quest for effective methods to treat hair dye effluents. Researchers have explored crucial tools to address persistent organic compounds, from advanced adsorption strategies to innovative processes such as biodegradation, bioremediation, and ozone-assisted techniques. These innovations contribute significantly to the comprehensive and sustainable management of pollutants in our wastewater, promoting a responsible and environmentally conscious approach to treating these residues. Table 1 summarizes key parameters and results from the articles discussed in this section.

**Table 1:** Parameters Applied for Different Treatment Methods for Dye Decontamination

treatment technique	dyes	adsorbent/catalyst/photocatalyst/	adsorption capacity (mg g^–1^)	% degradation and time of treatment	TOC or COD removal	*k* (min^–1^)	ref.
adsorption	AR, AY, and AE 50 mg L^–1^	oak cupules powder with ZnO	55.5 for AR 52.6 for AY 135.1 for AE	97% for AR, 120 min 77% for AY, 150 min 87% for AE, 120 min		0.0055 for AR 0.0084 for AY 0.0269 for AE	^161^
adsorption	BB16 15 mg L^–1^	durian shell adsorbent		77.6% in 30 min	80.6% in 30 min		^162^
adsorption	GARNIER color naturals (3.16 burgundy)	carbon hybrid magnetic nanosheets from the carbonization of bagasse	86.3	92.6% in 20 min			^163^
biodegradation	PPD	dye-degrading bacteria-I (DDB I)		100% in 12 h			^164^
bioremediation	hair dyeing wastewater	*Enterobacter cloacae* associated with sugar cane bagasse powder		85.23% in 18 h			^58^
PC	Basic Red 51 in hair dye greywater 7.48 mg L^–1^	ZnO NPs		82.7% in 300 min	72.2% in 300 min		^166^
PEC	Basic Red 51 Basic Blue 99 20 mg L^–1^	Ti/TiO_2_/Sb_2_S_3_ composite electrode		70% for BR51, 20 min 100% for BB99, 20 min	69% in 120 min	0.0404	^175^
PEC	Acid Yellow 1 100 mg L^–1^	boron-doped TiO_2_ nanotubes		95% in 60 min	95% in 120 min	0.0235	^171^
PEC	Basic Red 51 1.0 × 10^–5^ mol L^–1^	W/WO_3_ thin film		100% in 60 min	63% in 60 min	0.064	^169^
PEC	Basic Red 51 3.3 × 10^–5^ mol L^–1^	W/WO_3_/TiO_2_ bicomposite		100% in 60 min	94% in 120 min	0.066	^170^
PEC	Basic Brown 16 Basic Blue 99 3.3 × 10^–5^ mol L^–1^	W/WO_3_ nanopores		100% for BB16, 120 min 100% for BB99, 120 min	59% for BB16, 120 min 44% for BB99, 120 min	0.0329, BB16 0.0128, BB99	^46^
catalytic ozonation	Basic Yellow 87 216 mg L^–1^	porous copper fiber loaded with Cu/Zn/Al/Zr		100% in 4 h	COD: 60% in 240 min TOC: 30% in 240 min	0.028	^172^
O_3_/PEC	Acid Yellow 1 100 mg L^–1^	TiO_2_ nanotubes		99% in 9 min	100% in 60 min	0.156	^173^
O_3_/PEC O_3_/UV/H_2_O_2_ O_3_/PEC/H_2_O_2_	hair dyeing wastewater (dark brown color)	TiO_2_ nanotubes		100% in 60 min	58.7% in 90 min 87.7% in 90 min 92.4% in 90 min		^61^
PC—flocculation	greywater from permanent black hair dye	TiO_2_ (AEROXIDE P25), chitosan			TOC: 22% in 240 min PC 83% after flocculation, 24 h COD: 45% in 240 min PC 90% after flocculation, 24 h		^174^

## 6 Conclusions, Challenges, and Future Perspectives

The growing popularity and widespread use of hair dyes have underscored the importance of understanding and addressing the potential risks of toxicity and contamination. Research indicates that the components found in hair dyes may pose risks to human health and the environment. These risks include skin sensitization, potential carcinogenic effects, and negative pregnancy outcomes. Additionally, these dyes entering aquatic ecosystems via wastewater can contaminate water and harm aquatic organisms, underscoring the need for environmentally safe disposal methods.

Despite ongoing research, significant gaps remain in understanding hair dye toxicity, particularly regarding long-term exposure to low doses in environmental and human health contexts. Conflicting findings in epidemiological studies, particularly around cancer risk, reflect the complexity of establishing clear causal links across diverse exposure scenarios. Addressing these knowledge gaps will require further research, especially longitudinal studies that consider both direct and indirect exposure pathways.

The expansion of the hair dye industry has simultaneously fueled the development of sophisticated analytical techniques. While HPLC and GC offer excellent sensitivity and precision, they require significant technical expertise and expensive equipment and often produce environmentally harmful waste. Emerging electroanalytical methods, including modified electrode techniques, show considerable promise due to their sensitivity, affordability, and low environmental impact, although they currently face challenges related to selectivity.

Looking ahead, improving detection methodologies for hair dye contaminants remains a critical priority. A multidisciplinary approach involving toxicologists, environmental scientists, and analytical chemists is essential to developing safer hair dye formulations and effective waste treatment processes. Future research should prioritize scalable and cost-effective detection methods that minimize environmental impact while ensuring accurate monitoring of hair dye residues in various matrices. Collaboration between regulatory bodies and industry stakeholders will be vital in advancing safer hair dye products and implementing sustainable disposal practices to safeguard human health and the environment.

Substantial progress has been made in developing effective wastewater treatment methods to address the environmental impact of hair dye contaminants. AOP techniques, such as PEC treatments and ozonation, have demonstrated practical solutions for treating complex wastewater, achieving high levels of decolorization, mineralization, and contaminant removal and demonstrating success in mitigating the hazardous effects of hair dye effluents. Adsorbents derived from natural materials, such as oak cupules and agricultural waste, have shown significant potential for hair dye removal, particularly when combined with innovations in AOPs. Additionally, bacterial degradation methods for detoxifying toxic compounds like PPD have yielded promising results, reducing toxicity and environmental impact. Despite considerable advancements, challenges remain in the treatment of real effluents. The complex compositions and limited transparency of these effluents can reduce the treatment efficiency. Furthermore, while some adsorbents have demonstrated impressive reusability, maintaining long-term performance and preventing secondary pollution from degraded adsorbents remain areas needing improvement. Achieving consistently high efficiency while minimizing the environmental impact and operational costs in large-scale applications is a persistent challenge.

Future research should prioritize optimizing these methods to treat real-world, high-complexity effluents, including using renewable energy sources for processes such as photocatalysis and electrochemical treatments. Integrating treatment methods, such as combined PEC and ozonation, represents a promising approach to enhancing the decolorization and mineralization efficiency. Another promising direction is exploring novel catalysts and adsorbents with high stability, reusability, and a minimal environmental footprint. Additionally, the in situ generation of hydroxyl radicals and biobased detoxification processes could support safer and more cost-effective approaches.

In short, the expanding hair dye industry provides opportunities to gain deeper insights into hair structure, dyeing techniques, and their interactions. At the same time, it is responsible for addressing the environmental and health impacts associated with hair dye contaminants. Advances in treatment methods and detection techniques hold substantial promise, yet balancing efficiency, scalability, and environmental sustainability remains a significant challenge. Future progress will rely on collaboration among scientists, industry leaders, and regulatory bodies to enhance safety across all aspects—from product formulation to wastewater treatment. Prioritizing multidisciplinary approaches that integrate eco-friendly practices and renewable resources will be critical for minimizing risks to humans and the environment while fostering a safer and more sustainable industry.

## Supplementary Material



## Notes

Molecular absorption spectroscopic data of various hair dyes and their associated toxicity, and analytical methods for determining temporary, semipermanent, and permanent dyes and their derivatives in different matrices (PDF)
