# Supplementary material for: Comprehensive Review of Hair Dyes: Physicochemical Aspects, Classification, Toxicity, Detection, and Treatment Methods
Source: ACS Omega. 2025 Jun 27;10(27):28567–86. doi: 10.1021/acsomega.5c01576 (PMC12268732; doi:10.1021/acsomega.5c01576)
Supplement: Supplementary file 1 [file ao5c01576_si_001.pdf]

SUPPORTING INFORMATION

COMPREHENSIVE REVIEW OF HAIR DYES: PHYSICOCHEMICAL ASPECTS, CLASSIFICATION, TOXICITY, DETECTION AND TREATMENT METHODS

João Carlos de Souza[a,b], Elisa Raquel Anastácio Ferraz Avelino[c], Guilherme Garcia Bessegato[d], Rogério do Carmo Gonçalves da Costa[e], Patricia Alves Carneiro[e], Danielle Palma de Oliveira[c], Maísa Azevedo Beluomini[b,f], Juliana Ferreira de Brito[b*] and Maria Valnice Boldrin Zanoni[b]

[a]University of São Paulo (USP), Faculty of Philosophy, Sciences, and Letters at Ribeirão Preto (FFCLRP), Department of Chemistry
Avenida Bandeirantes, 3900 - 14040-901 - Ribeirão Preto, São Paulo State, Brazil

[b]São Paulo State University (UNESP), Institute of Chemistry, Department of Analytical, Physical-Chemical and Inorganic Chemistry
Rua Professor Francisco Degni, 55, Araraquara - 14800-060, São Paulo State, Brazil

[c]University of São Paulo (USP), Faculty of Pharmaceutical Sciences at Ribeirão Preto
Avenida Bandeirantes, 3900 - 14040-901 - Ribeirão Preto, São Paulo State, Brazil.

[d]Universidade Tecnológica Federal do Paraná (UTFPR), Dois Vizinhos Campus
Estrada para Boa Esperança km 04 - 85660-000 - Dois Vizinhos, Paraná State, Brazil

[e]Federal Fluminense University (UFF), Institute of Exact Sciences (ICEx), Department of Chemistry
Rua Desembargador Ellis Hermydio Figueira, s/n - 27213-145 - Volta Redonda – Rio de Janeiro State, Brazil

[f]São Paulo State University (UNESP), School of Agricultural and Veterinary Sciences
Via de Acesso Prof. Paulo Donato Castellane s/n - 14884-900 - Jaboticabal, São Paulo State, Brazil

*Correspondent Author
E-mail: Juliana.f.brito@unesp.br
Phone: + 55 16 3301 9618

Table S1. Molecular absorption spectroscopic data of various hair dyes and their associated toxicity.

Dye	UV-Vis Spectra	Spectra data	Toxicity	Ref.
**Acid Orange 7**	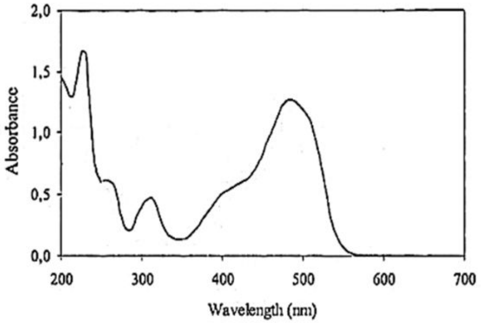	λ ≈ 484 nm in aqueous or neutral solutions for **π→π*** transitions involving the azo group (–N=N–) and conjugated aromatic rings.	May lead to organ damage following prolonged or repeated exposure and poses a long-term hazard to aquatic ecosystems.[38,39]	40
**Acid Red 33**	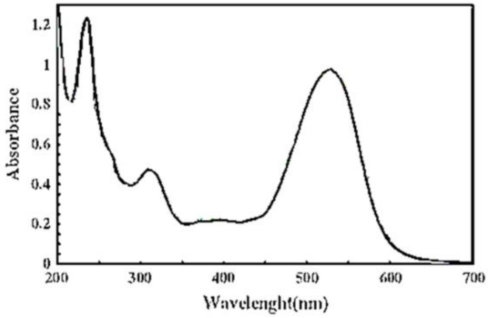	λ ≈ **507 nm** in aqueous solution for **π→π*** transitions involving the azo group (–N=N–) and aromatic rings.	No hazards have been identified or classified based on company submissions under REACH registrations to ECHA. Based on GHS Hazard Statements, it causes severe damage, eye irritation, and Harm to aquatic life with long-lasting effects.[41,42]	43

**Basic Blue 99**	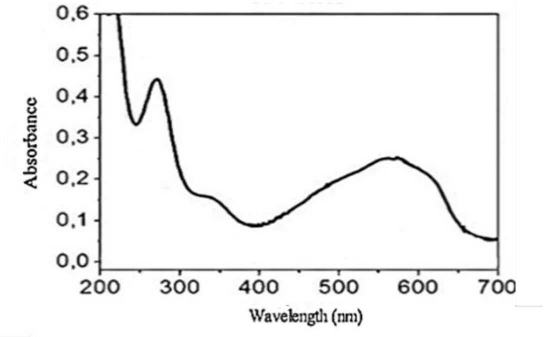	**λ ≈ 270 nm** corresponds to π→π* transitions within the aromatic rings.<br><br>**λ ≈ 577 nm** associated with the extended conjugated system.<br><br>**λ ≈ 619 nm** linked to the chromophoric system.	Based on CLP notifications submitted by companies to ECHA and GHS Hazard, may cause serious eye damage/eye irritation. [44,45]	46

**Basic Brown 16**	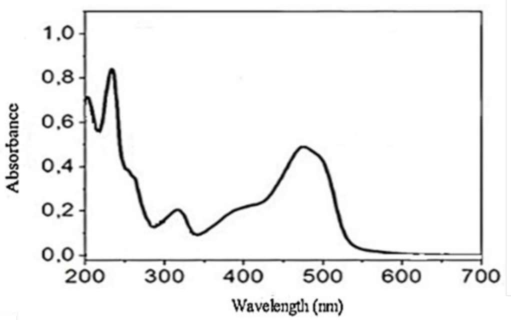	**λ ≈ 218 nm** corresponds to π→π* transitions within the aromatic rings.<br><br>**λ ≈ 259 nm** associated with n→π* transitions, typical for azo compounds.<br><br>**λ ≈ 478 nm** attributed to the extended conjugated system.	Based on the REACH registration data submitted to ECHA and GHS Hazard Statements, this substance is considered a potential skin sensitizer and may trigger allergic reactions on contact and serious eye damage/irritation. [47,48]	[46]

**Acid Yellow 1**	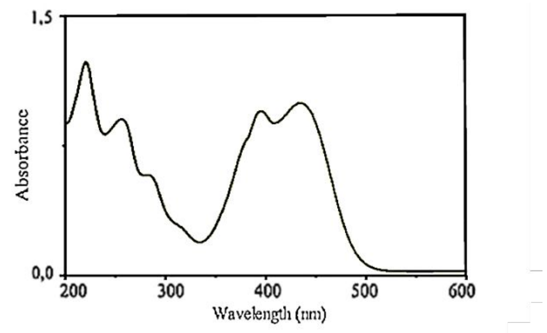	**λ ≈ 392 nm** corresponds to π→π* transitions within the aromatic rings.<br><br>**λ ≈ 428 nm** associated with the azo (–N=N–) chromophore.	CLP notifications submitted by companies to ECHA indicate that this substance may cause organ damage with prolonged or repeated exposure and could lead to allergic skin reactions. Based on GHS Hazard Statements<br>Prolonged or repeated exposure may result in organ damage Specific target organ toxicity – repeated exposure.[49,50]	51
**Acid Red 17**	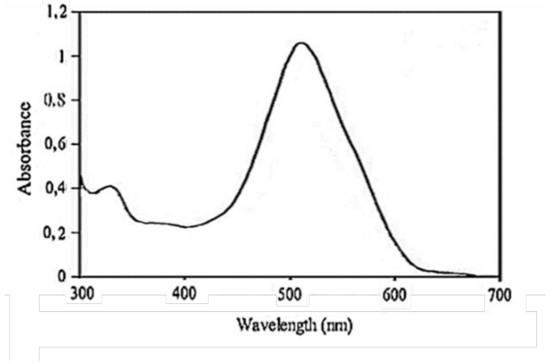	**λ ≈ 518 nm** in aqueous solution associated with the azo (–N=N–) chromophore.	Based on CLP notifications submitted by companies to ECHA and GHS Hazard Statements, this substance is classified as causing serious eye irritation and skin irritation.[52]	53

**Acid Yellow 3**	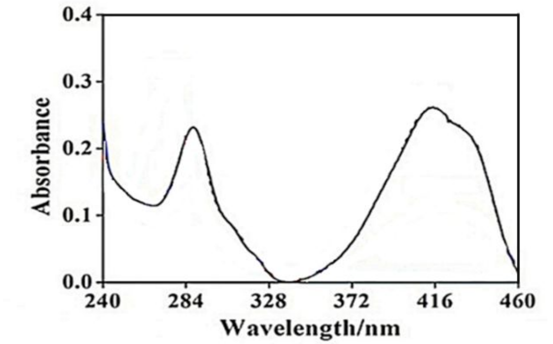	**λ ≈ 416 nm** in aqueous solution associated with the azo (–N=N–) chromophore.	No hazards have been identified or classified based on company submissions under REACH registrations to ECHA and GHS Hazard Statements, no data is available. Nonetheless, large or frequent releases into the environment could have harmful effects.[54]	55
**Basic Red 26**	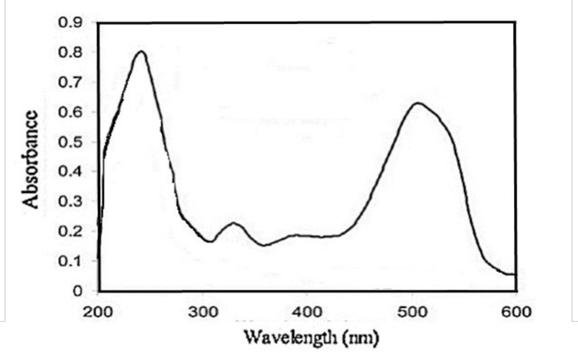	**λ ≈ 352–360 nm** corresponds to π→π* transitions within the aromatic rings of the dye molecule.	No data is available under REACH registrations to ECHA or COMPTOX (EPA). Nonetheless, large or frequent releases into the environment could have harmful effects.[56]	57

Table S2. Analytical methods for determining temporary, semi-permanent, and permanent dyes and their derivatives in different matrices.

Sample	Analyte	Analytical Technique	Limit of Detection	Limit of Quantification*	Linear range	Recovery (%)	Ref.
Hair dyes and water	PPD and RSN	LWS/GCE/MWNTs–CHT	0.79 and 0.58 mg L^{-1}	2.66 and 1.87 mg L^{-1}	0.55 - 21.2 mg L^{-1}	97.1 -102.2	104
Wastewater and water	BB Derivative	LWS/GE	0.04 mg L^{-1}	0.1 mg L^{-1}	0.1 - 1.0 mg L^{-1}	95.9 – 98.1	60
Biological fluids	PTD and PAP	DPV/GCE	1.2 - 3.9×10^{-7} mol L^{-1}	1.7- 4.6×10^{-7} mol L^{-1}	0.1 -1.0×10^{-6} mol L^{-1}	96.7 – 98.3	105
Wastewater and water	PTD and PAP	HPLC-DAD	0.02 and 0.03 mg L^{-1}	0.07 and 0.08 mg L^{-1}	0.01 - 500.0 mg L^{-1}	95.1 - 97.1	30
Effluent and Water	Red 1, Violet 93, Blue 373, Orange 1, Orange 3, Orange 25, Yellow 3, Yellow 7 and Red 13	SPE-LC-ESI-MS/MS	0.5 ng L^{-1}	2.0 ng L^{-1}	2.0 - 100.0 ng mL^{-1}	68.0 – 115.0	112
Hair dyes	Basic Blue 26, Basic Blue 99, Basic Red 76, Basic Brown 17, Disperse Blue 1, Disperse Blue 3, Disperse Violet 1,	HPLC-DAD	1.0 – 5.0 µg mL^{-1}	-	10.0 – 100.0 mg mL^{-1}	96.8 – 102.4	118

	Melange Acid Black and Anthraquinone						
Biological fluids and water	PTD	SWV/CSPE/MNP-CA-CYS	8.53×10^{-8} mol L^{-1}	2.56×10^{-7} mol L^{-1}	8.0×10^{-7} - 8.0×10^{-5} mol L^{-1}	97.5 – 105.0	[121]
Human blood	PPD and metabolites	LC–MS/MS	-	-	10.0 – 2000.0 ng mL^{-1}	51.9 – 56.2	[123]
Hair dyes, wastewater, and water	Basic Red 51	SWV/GECE/CFMNs	1.19×10^{-7} mol L^{-1}	3.97×10^{-7} mol L^{-1}	1.5×10^{-6} - 5.5×10^{-5} mol L^{-1}	96.4 - 103.7	[124]
Urine	PPD and metabolites	MALDI-MS/MS	-	5.0×10^{-5} mol L^{-1}	0.05 – 1.0 mol L^{-1}	7.7 – 48.9	[125]
Hair dyes	2,6-Diaminopyridine	CV/DICSN–graphene–IL	0.028 mg kg^{-1}	-	0.050 - 35.0 mg kg^{-1}	98.4 – 103.8	[126]
Wastewater and water	Acid Green 25	SWV/GCE/MWCNTs	2.7×10^{-9} mol L^{-1}	8.9×10^{-9} mol L^{-1}	1.0×10^{-7} - 7.0×10^{-6} mol L^{-1}	96.7	[127]
Hair dyes, henna, and dyed hair	Aromatic amines	GC–MS	0.02 - 0.20 ng g^{-1}	-	10.0 - 100.0 ng L^{-1}	92.2 - 98.4	[128]
Hair dyes	Aromatic amines	HPLC-ED	1.5 – 4.0 ng mL^{-1}	-	0.3 – 30.0 µg mL^{-1}	96.0 – 100.0	[129]
Water	Aromatic amines	SPME/HPLC-UV	0.5 – 16.9 ng mL^{-1}	-	5.0 – 500.0 ng mL^{-1}	55.3 – 104.7	[130]

Pharmaceutical formulations	Acetaminophen and PAP	mini-CE-AD	5.9×10^{-7} and 1.4×10^{-6} mol L^{-1}	-	$5.0\times10^{-6} – 1.0\times10^{-3}$ mol L^{-1}	97.1 and 98.3	131
Wastewater	Basic Blue 9, Basic Brown 16, Basic Red 76, Basic Yellow 57, and Acid Violet 43	LC-MS/MS-SRM	0.66 - 63 ng mL^{-1}	-	1.0 to 200.0 ng mL^{-1}	79.0 – 98.0	132
Hair dyes and water	Basic Blue 99, Acid Violet 43, Basic Brown 16, Basic Red 76, and Basic Yellow 57	IL-HPLC-DAD	$0.53 - 2.98\times10^{-7}$ mol L^{-1}	$1.08 - 3.66\times10^{-7}$ mol L^{-1}	$2.97\times10^{-7} - 1.50\times10^{-5}$ mol L^{-1}	83.0 – 100.0	133
Hair dyes	PPD	GC-MS	0.05 mg mL^{-1}	-	-	97.5 – 99.1	134
Hair dyes	*o,m,p*-phenylenediamine, Catechol and RSN	CZE–AD	$0.18 – 1.57\times10^{-6}$ mol L^{-1}	-	$1.0\times10^{-6} - 1.0\times10^{-4}$ mol L^{-1}	91.0 – 108.0	135
Hair dyes	PPD, 4-chlororesorcinol, and *m*-aminophenol	SWV/GCE	$0.6 – 1.2\times10^{-6}$ mol L^{-1}	-	$2 - 200\times10^{-7}$ mol L^{-1}	-	136
Wastewater	Basic Brown 16	SWV/GECE/CFMNs	1.01×10^{-8} mol L^{-1}	2.37×10^{-8} mol L^{-1}	$1.0\times10^{-7} - 1.0\times10^{-6}$ mol L^{-1}	99.1 – 102.4	137
Wastewater	Basic Blue 41	SWV/SPCE/Gr	5.0×10^{-9} mol L^{-1}	1.7×10^{-8} mol L^{-1}	$3.0\times10^{-8} - 2.01\times10^{-6}$ mol L^{-1}	98.5 - 102.4	138

Water	Basic Brown 17	LWV/ Ti/TiO_2 Nanotube	1.3×10^{-7} mol L^{-1}	4.4×10^{-7} mol L^{-1}	1.0×10^{-6} - 8.0×10^{-5} mol L^{-1}	99.4	[139]
Wastewater	PAP and *o*-aminophenol	UV-Vis spectrophotometry	0.010 µg cm^{-2}	-	2.18 – 9.28 µg mL^{-1}	98.1 – 99.9	[140]
Water	PAP	CuO/H-Gr	0.69 and 0.01×10^{-6} mol L^{-1}	-	1.0 - 200 and 0.01–300×10^{-6} mol L^{-1}	-	[141]
Hair dyes	Aromatic amines	IL-HPLC-ED	0.021 - 0.246 mg L^{-1}	0.303 – 2.021 mg L^{-1}	1.09 - 217.0 mg L^{-1}	95.0 – 103.0	[142]
Water	PAP	Fe_3O_4@Au/MOF peroxidase-like	0.38×10^{-6} mol L^{-1}	-	0.1 – 10×10^{-6} mol L^{-1}	98.6 - 105.3	[143]
Water	PAP, phenol and *p*-nitrophenol	DPV/CPE/PDA-Iodine	30, 40 and 80×10^{-9} mol L^{-1}	-	0.5 – 120×10^{-6} mol L^{-1}	94.0 – 104.4	[144]
Hair dyes, wastewater, and water	Acid Green 25	DPV/IL/$CoFe_{12}O_{19}$/GO/CPE	0.067×10^{-6} mol L^{-1}	-	0.08 - 900×10^{-6} mol L^{-1}	97 - 106	[145]
Hair dyes	PPD	DPV/MB/N-CoNi/C/GCE	0.091×10^{-6} mol L^{-1}	-	0.3 – 100×10^{-6} mol L^{-1}	95.4 – 107.3	[146]
Hair dyes and biological fluids	RSN	DPV/M/MWCNT/MCPE	0.02×10^{-6} mol L^{-1}	-	0.5 – 10×10^{-6} mol L^{-1}	97.5 – 101.8	[147]
Hair dyes, cream, and water	Hydroquinone, catechol, and RSN	DPV/GCE/N-NiCSs	0.002, 0.02 and 0.24×10^{-6} mol L^{-1}	-	0.005–500.0×10^{-6} mol L^{-1}	92.3 – 108.2	[148]
Hair dyes, hair, and percutaneous absorption	*o*-phenylenediamine, *m*-phenylenediamine, PPD and PTD	MEKC-LIF	25 - 100×10^{-9} mol L^{-1}	-	0.125 – 5.0×10^{-6} mol L^{-1}	99.0 – 108.0	[149]

Hair care products	26 oxidative hair dye ingredients	GC–MS	0.02 – 2.58 µg g^{-1}	0.05 – 7.75 µg g^{-1}	0.1 – 100.0 µg g^{-1}	-	[150]
Hair dyes	40 oxidative hair dye ingredients	HPLC-DAD	5.0 - 168.0 µg g^{-1}	16.0 - 504.0 µg g^{-1}	5.0 - 500.0 mg L^{-1}	81.4 - 109.6	[151]
Hair dyes	22 oxidative hair dye ingredients	GC-MS	15.0 - 35.0 µg g^{-1}	47.0 - 115.0 µg g^{-1}	5.0 - 500.0 mg L^{-1}	89.2 - 103.2	[152]
Hair Dyes	54 oxidative hair dye ingredients	LC-HRMS	0.1 – 23.5 ng mL^{-1}	0.2–78.1 ng mL^{-1}	-	60.0 - 118.4	[153]
Hair Dyes products	11 hair dyes	LC–MS/MS	0.15 - 10.0 mg kg^{-1}	0.5 - 40.0 mg kg^{-1}	-	79.4 - 109.2	[154]
Swab, stratum corneum, skin, and receptor fluid	Gallic acid	HPLC-UV-VIS	-	0.5 - 30 µg mL^{-1}	-	90.3 – 112.8	[155]
Swab wash, stratum corneum, skin, and receptor fluid	4-amino-3-nitrophenol	LC-MS/MS	50.0 and 80.0 ng mL^{-1}	-	20.0 – 1000.0 ng mL^{-1}	93.5 – 111.7	[156]

Hair dyes and cosmetics	PPD	RP-HPLC-DAD	0.025% (w/v)	0.10% (w/v)	20.0 – 1000.0 mg L^{-1}	83.0 - 94.4	[157]
Hair dyes	PPD	UV-Vis spectrophotometry	0.2445 µg mL^{-1}	0.7411 µg mL^{-1}	2.0 – 10.0 µg mL^{-1}	98.9 - 100.1	[158]
Wash, swab, stratum corneum, skin, and receptor fuid	2-amino-5-nitrophenol	HPLC-UV-VIS	-	0.5 µg mL^{-1}	0.5 - 50.0 µg mL^{-1}	93.1–110.2	[159]
Hair dyes	PPD	MB/N-CoNi/C/GCE	0.091×10^{-6} mol L^{-1}	-	0.3 – 100.0×10^{-6} mol L^{-1}	95.1 - 107.3	[146]
Hair dyes	Hydroquinone (HQ), catechol (CC), and RSN	N-NiCSs/GCE	1.52×10^{-9} mol L^{-1} for HQ<br>1.50 $\times10^{-8}$ mol L^{-1} for CC<br>2.4×10^{-7} mol L^{-1} for RSN	-	0.005 – 100×10^{-6} mol L^{-1} for HQ<br>0.05 – 200×10^{-6} mol L^{-1} for CC<br>5.0 – 500×10^{-6} mol L^{-1} for RSN		[148]
Hair dyes	PPD	S-PPD-DCM	5.35 µg L^{-1}	-	10 – 100 µg L^{-1} and 100 – 1000 µg L^{-1}	94.7 - 98.1	[160]

*Only the limits of quantification presented in the articles were reported in the table.
